# Stress‐Resistant Symbiodiniaceae and Diverse Bacterial Communities Promote Coral Persistence in Variable, Multi‐Stressor Environments

**DOI:** 10.1002/ece3.73783

**Published:** 2026-06-02

**Authors:** Maya E. Powell, Sarah L. Solomon, Verena Schoepf, Karl D. Castillo

**Affiliations:** ^1^ Environment, Ecology, and Energy Program University of North Carolina at Chapel Hill Chapel Hill North Carolina USA; ^2^ Department of Freshwater and Marine Ecology, Institute for Biodiversity and Ecosystem Dynamics University of Amsterdam Amsterdam the Netherlands; ^3^ Department of Earth, Marine and Environmental Sciences University of North Carolina at Chapel Hill Chapel Hill North Carolina USA

**Keywords:** bacteria, biodiversity, coral, microbiome, natural laboratory, Symbiodiniaceae

## Abstract

Coral holobionts maintain complex symbioses, which can be influenced by global and local stressors. However, the role of coral‐associated bacterial and algal (Symbiodiniaceae) communities in facilitating coral persistence in variable and extreme, multi‐stressor environments is not fully understood. Multi‐stressor environments, such as semi‐enclosed inland bays, provide natural laboratories to study potential coral holobiont responses to future ocean conditions in situ. We investigated the role of Symbiodiniaceae and bacterial communities in coral holobiont persistence across habitats and dry versus wet seasons. We collected three Caribbean coral species (*Siderastrea siderea, Siderastrea radians,* and branching *Porites* sp.) from two semi‐enclosed inland bays and two nearby fringing reefs in Curaçao across three seasonal timepoints. We show that all coral species hosted high proportions of stress‐tolerant Symbiodiniaceae in inland bays, likely facilitating the survival of their coral hosts in these turbid and highly variable and extreme environments. We also observed distinct differences in bacterial community composition across habitats, sites, and seasons in *S. siderea* and *S. radians* but not branching *Porites* sp. Bacterial communities of *S. siderea* and *S. radians* contained higher proportions of bacteria with the potential for diverse metabolisms, such as sulfur and nitrogen cycling, in inland bays and during wet seasons. Environmental conditions therefore strongly influence bacterial community composition, and bacterial diversity and metabolic flexibility are likely critical for coral holobiont success across environments and seasons. Our findings show how Caribbean coral holobionts persist in multi‐stressor environments and may respond as anthropogenic climate change continues to exacerbate and intensify these stressors.

## Graphical abstract

Corals living in multi‐stressor bays in Curaçao maintain stress‐tolerant symbiotic algae and specialized bacterial communities in comparison to those in nearby reefs. These findings provide insight into how Caribbean coral holobionts persist in extreme and marginal conditions and may respond under future environmental conditions.

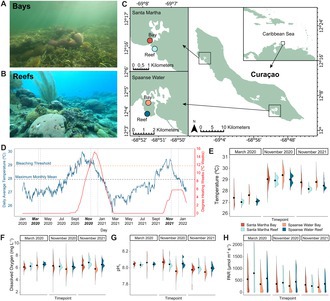

## 1 Introduction

Coral reefs are declining worldwide due to the combined effects of climate change and local anthropogenic stressors (IPCC 2023). Acute and chronic stressors at the climate, regional, and local level can result in degraded environmental and ecological conditions, leading to regime shifts towards non‐coral dominated states and reduced ecosystem functioning (Bell et al. 2021; Haas et al. 2016; Harborne et al. 2017). The ability to survive under changing conditions depends upon complex interactions between coral and their associated Symbiodiniaceae and bacterial partners. These partners may facilitate holobiont acclimatization and persistence under multiple stressors (Camp et al. 2019). Corals are holobiont organisms, comprising the host skeleton and tissue, dinoflagellate algal symbionts (family Symbiodiniaceae), bacteria, fungi, archaea, viruses, and more (Rohwer et al. 2002). Each of these members display different functional traits and plasticity within these traits, potentially conferring benefits to the holobiont, such as nutrient cycling and thermotolerance, across a range of environmental conditions and time scales (Voolstra and Ziegler 2020). Coral‐associated microbial and algal communities are able to respond to environmental changes on time scales of hours to months, providing a potential key mechanism for holobiont resilience under a rapidly changing climate and intensifying local stressors (Caughman et al. 2021; Voolstra and Ziegler 2020). Despite the crucial roles of bacterial and symbiont communities in promoting coral health and survival on shorter time scales, their capacity to facilitate resilience under multiple stressors is not fully understood.

Symbiodiniaceae, in particular, are essential members of the coral holobiont as they provide the coral host with carbon needed for energy, growth, reproduction, and more (LaJeunesse et al. 2018; Muscatine and Porter 1977). Additionally, Symbiodiniaceae with beneficial traits such as higher carbon uptake, increased photochemical efficiency, and thermotolerance can promote holobiont survival (Cunning et al. 2018; Ros et al. 2021; Silverstein et al. 2015). For example, symbiont species *Durusdinium trenchii* is known to be thermotolerant, and can increase coral bleaching thresholds by ~1°C–1.5°C, while the genus *Cladocopium* is very biodiverse, containing both heat sensitive as well as thermotolerant species (Davies et al. 2023). Coral‐Symbiodiniaceae coevolutionary relationships can create species‐specific symbioses, however, the ability to associate with a range of Symbiodiniaceae species can also augment coral survival and resilience via increased acclimatization capacity (Forsman et al. 2020; Putnam et al. 2012; van Oppen and Medina 2020). As such, corals can shuffle or switch their symbiont communities to stress‐resistant Symbiodiniaceae taxa to combat environmental stress and potentially survive bleaching, a process defined as the significant loss of symbionts and/or photosynthetic pigments due to heat or other abiotic stress (Hoegh‐Guldberg and Smith 1989). Symbiont shuffling occurs via proliferation of small populations of Symbiodiniaceae already within the coral tissue and switching by acquiring new symbionts from nearby corals, the water column, and surrounding sediment (Cunning et al. 2015; Kinzie et al. 2001; Williamson et al. 2021). Additionally, holobiont thermal tolerance may also be conferred through local adaptation of symbiont types to their thermal environment (Howells et al. 2016). In addition to thermotolerance, Symbiodiniaceae can also facilitate tolerance to suboptimal environmental conditions, such as lower light, salinity, pH, and more, providing them, and their coral hosts, with tolerance to multiple stressors (LaJeunesse 2002; Nitschke et al. 2022).

Coral‐associated bacterial community restructuring and environmental specificity is another adaptive strategy that may aid in coral survival under extreme environmental conditions. Bacteria affect holobiont nutrient cycling and influence coral disease resistance and health through mechanisms such as providing fixed nitrogen to the coral host and generating antibacterial compounds (Peixoto et al. 2017; Pogoreutz et al. 2021; Rädecker et al. 2015; Voolstra et al. 2024). Furthermore, recent work on coral probiotics has identified beneficial microorganisms for corals (BMCs) that can mitigate coral bleaching and aid in their survival (Osman et al. 2025; Peixoto et al. 2021; Santoro et al. 2019). Bacterial community changes can also occur over periods as short as a few hours, and may provide a potential mechanism of resilience in highly fluctuating environments (Camp et al. 2020; Caughman et al. 2021; Kriefall et al. 2022; Sweet et al. 2017; Voolstra and Ziegler 2020). Mechanisms behind these rapid community shifts are still not fully understood, although environmental conditions likely play a major role. Additionally, the coral host represents a potential biological control of their bacterial and Symbiodiniaceae communities via holobiont nutrient cycling and host species‐specificity (Chu and Vollmer 2016; Howells et al. 2020; Matz 2024; Morrow et al. 2012). Thus, characterizing bacterial and Symbiodiniaceae communities across environmental gradients, seasons, and coral species is important for determining holobiont dynamics, predicting future holobiont assemblages, and identifying potential resilient bacteria and holobiont communities. Extreme reef environments where coral communities live at the edges of their environmental and ecological limits can provide natural laboratories to study the effects of potential future ocean conditions on coral holobionts in situ (Camp et al. 2018; Maggioni et al. 2021). Although these natural laboratories are not perfect analogs for future ocean conditions, they provide the ability to study the effects of long‐term coral acclimatization to extreme, multi‐stressor conditions. Here, we define extreme and marginal coral communities according to the new framework developed by Schoepf et al. (2023) where extremeness describes abiotic conditions that differ greatly from optimal conditions, both in terms of mean and variability, while marginality describes ecosystem function and community composition that varies greatly from coral‐dominated, biodiverse reefs. Extreme and marginal conditions exist in semi‐enclosed inland bays in Curaçao (southern Caribbean) where coral communities experience highly variable and extreme abiotic conditions, along with low and patchy coral cover (< 2%) and low coral species diversity (de Jong et al. 2025; Debrot et al. 1998; Vermeij et al. 2007). The coral communities in the inland bays are severely understudied despite their potential to offer important insights into the mechanisms underlying coral multi‐stress tolerance, as has been shown recently in other extreme reef environments (Camp et al. 2019; Gantt et al. 2024; Grupstra, Meyer‐Kaiser, et al. 2024; Tanvet et al. 2023). These extreme environments often harbor corals with stress resistant Symbiodiniaceae (Ros et al. 2021) and specific, diverse bacterial assemblages (Camp et al. 2020). However, most of this work on extreme environments focuses on Pacific corals and Symbiodiniaceae communities, highlighting the need to assess corals in the Caribbean and to examine Symbiodiniaceae and bacterial communities in tandem. It is critical to determine how coral holobionts survive in these extreme and marginal conditions and to assess the potential roles of coral bacterial and symbiont communities in facilitating holobiont survival in multi‐stressor conditions.

Here, we utilized semi‐enclosed bays in Curaçao as natural laboratories to investigate bacterial and Symbiodiniaceae assemblages that may facilitate coral holobiont survival in extreme and marginal habitats. Specifically, we compared the diversity and community structure of Symbiodiniaceae and bacterial communities between extreme and more optimal coral habitats (bay vs. reef) across three seasonal timepoints for three Caribbean coral species with different morphologies and life history strategies: *Siderastrea siderea* , *Siderastrea radians* , and branching *Porites* sp. We hypothesized that bay corals would have higher proportions of stress‐resistant algal and bacterial taxa compared to conspecifics from fringing reefs. Additionally, we hypothesized that the abundance of these stress‐resistant taxa would increase during the warm, wet season compared to the cooler, dry season. Lastly, we posited that *S. siderea* , *S. radians* , and branching *Porites* sp. would display distinct bacterial and symbiont communities based on their morphological and life history differences.

## 2 Materials and Methods

### 2.1 Site Characterization

**FIGURE 1:**
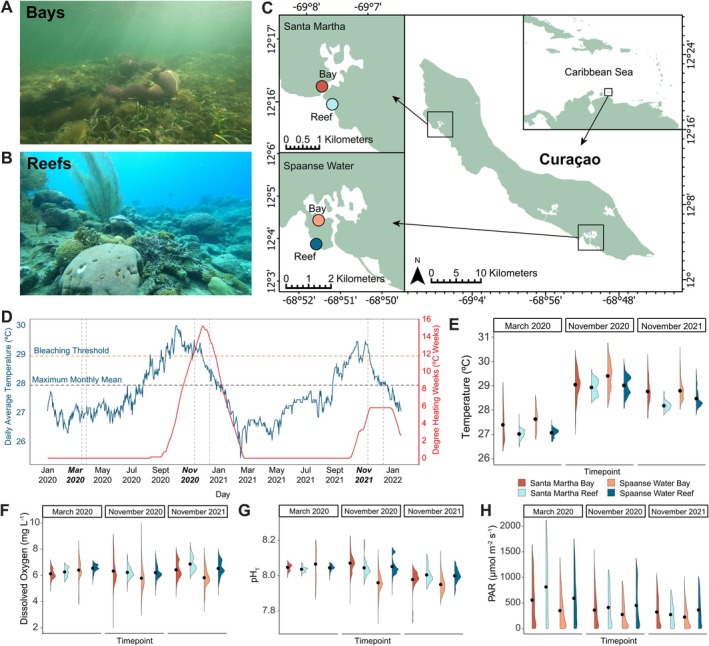
(A) Photo of an extreme and marginal inland bay in Curaçao. (B) Photo of a clear‐water fringing reef in Curaçao. (C) Map of Curaçao with sampling sites highlighted with colored dots and labels in the left panel: Santa Martha Bay (dark orange) and Spaanse Water Bay (light orange), Santa Martha Reef (light blue) and Spaanse Water Reef (dark blue). (D) Daily average temperature (°C, blue) and cumulative degree heating weeks (°C‐weeks, red) from January 2020 through January 2022. Dotted horizontal lines represent the local maximum monthly mean temperature (black) and bleaching threshold (orange) temperatures. Coral collection timepoints are denoted in bold, italicized text (March 2020, November 2020, November 2021). (E–H) Half violin plots display the distribution and range of the data where points represent the mean value for (E) Temperature (°C). (F) Dissolved oxygen (mg L^−1^). (G) pH_T_ and (H) Photosynthetically Active Radiation (PAR, μmol m^−2^ s^−1^). Data from March and November 2020 from de Jong et al. (2025). Statistical comparisons of daily averages for all environmental data can be found in Table S1.

The island of Curaçao is located in the Southern Caribbean and has fringing reefs along the southeast coast of the island as well as several inland bays that connect to the ocean via narrow channels (Figure 1A,B) (Vermeij et al. 2007). We selected four sites for this study, comparing two semi‐enclosed inland bays: Santa Martha Bay (12°16′10.10″ N: 69°7′45.01″ W) and Spaanse Water Bay (12°4′15.35″ N: 68°51′30.61″ W), to two nearby fringing reefs: Santa Martha Reef (12°15′59.56″ N: 69°7′39.66″ W), and Director's Bay (12°3′53.48″ N: 68°51′36.69″ W), hereafter referred to as Spaanse Water Reef (Figure 1C). The benthic substrate at both bay locations consists of sand, silt, and rubble, with the only suitable hard substrate for coral recruitment resulting from limestone outcroppings and anthropogenic substrates such as concrete, pipes, and sunken boats (de Jong et al. 2025; Vermeij et al. 2007). Flora and fauna of the bays consist of a variety of fish, seagrasses (mainly *Thalassia testudinum* ), mangroves, sponges, *Diadema* sp., *Cassiopeia* sp., other invertebrates, and corals (Debrot et al. 1998; Kuenen and Debrot 1995; Nagelkerken et al. 2001; Vermeij et al. 2007). Coral species composition in the bays has shifted greatly over time in tandem with global change (de Jong et al. 2025; Roos 1964; Vermeij et al. 2007). Bays are marginal coral habitats (*sensu* Schoepf et al. 2023) and have patchy coral cover of less than 2%, coral diversity of less than 18 species, and only four in some locations, with very low species evenness (Roos 1964; Vermeij et al. 2007). Environmentally, bays are extreme (sensu Schoepf et al. 2023) and have multiple stressors that deviate from optimal conditions in magnitude and variability (de Jong et al. 2025).

Fringing reefs in Curaçao have some of the highest coral cover in the Caribbean, with high biodiversity and connectivity (Schmutz et al. 2017). In comparison, inland bays are extreme environments with higher and more variable temperatures as well as generally lower and more variable pH, dissolved oxygen, and photosynthetically active radiation (PAR) (Figure 1E–H). Benthic cover in the bays consists of no reef framework, but instead, isolated coral assemblages; mostly seagrass, macroalgae, mangrove beds, fine sediment, and rubble. This, in combination with long seawater residence times, likely drives high environmental variability, especially in pH and dissolved oxygen (de Jong et al. 2025). Additionally, the bays, especially Spaanse Water Bay, have large anthropogenic influence since they are directly adjacent to urban development, increasing exposure to inorganic nutrients and toxins via pollution from sewage input, runoff, and boating (de Jong et al. 2025).

Environmental data were collected from all four sites in March 2020 (cooler dry season) and November 2020 (warm, wet season with heat stress) as described in (de Jong et al. 2025), as well as November 2021 (warm, wet season with only minor heat stress) according to the same protocols (Solomon et al. 2025). This data included continuous measurements of temperature, conductivity and dissolved oxygen, alongside semi‐continuous measurements of photosynthetically active radiation (PAR) and pH (de Jong et al. 2025). Discrete water samples were opportunistically collected at all sites to assess inorganic nutrient concentrations (nitrate, ammonium, phosphate) were measured at all sites including total alkalinity (four sites, March 2020 and November 2020 only), sediment trap collection data (four sites, November 2020 only), ecotoxicological data on organic pollutants (four sites, November 2020 only), and carbon and nitrogen content and trace metal concentrations of seagrasses (two bays only, November 2020 only) (de Jong et al. 2025). Importantly, the bays maintained higher water‐column nutrient levels (nitrate, ammonium, and phosphate) compared to the reef, especially during the wet season (de Jong et al. 2025; Solomon et al. 2025). Furthermore, seagrass samples from the bays displayed high levels of nutrients and the presence of trace metals (de Jong et al. 2025). Extensive site characterization and environmental monitoring data can be found in de Jong et al. (2025) and Solomon et al. (2025).

For all environmental data, outlier values that exceeded three times the interquartile range were removed before processing. Kruskal–Wallis tests using the Scheirer‐Ray‐Hare extension (*scheirerRayHare*) were utilized to assess differences between daily averages of each environmental variable across habitats, timepoints, sites, habitat:timepoints, and site:timepoints (Mangiafico 2024). Levels were compared with post hoc Dunn tests (*dunnTest*) with Benjamini‐Hochberg *p*‐value corrections for multiple comparisons (Table S1) (Ogle et al. 2023).

Degree heating weeks (DHW) were calculated as a measure of cumulative heat stress using combined temperature data from dissolved oxygen (DO) loggers deployed at the two fringing reef study sites, Aqualink buoy data from CARMABI House reef (https://aqualink.org/sites/1150), and NOAA (Coral Reef Watch) satellite time series data (region: Aruba, Curaçao, and Bonaire). Since in situ temperature loggers were only deployed for certain time periods (Figure 1D), data sources were prioritized in this order based on data availability. The local Maximum Monthly Mean temperature (MMM) for the region: Aruba, Curacao, and Bonaire was used to calculate DHW and bleaching thresholds. We were unable to calculate DHW for the bays, since no historical records of temperatures or bleaching thresholds were available for either bay. Since the bays are ~0.8°C warmer than reefs, we expect that they have a higher MMM, and thus bleaching threshold, than the reefs (de Jong et al. 2025).

### 2.2 Coral Collection

**FIGURE A1:**
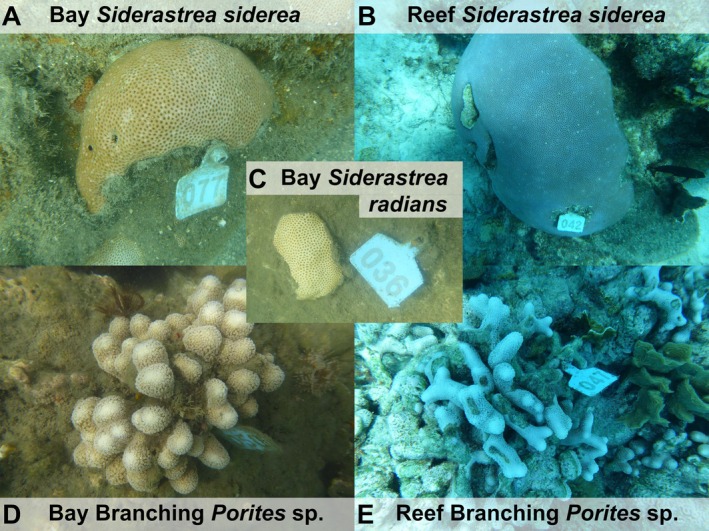
Coral species in inland bay and fringing reef sites. (A) Siderastrea siderea colony in an inland bay. (B) *S. siderea* colony in a fringing reef. (C) Siderastrea radians colony in an inland bay. *S. radians* was only sampled in inland bays for this study. (D) Branching Porites sp. colony in an inland bay site. (E) Branching Porites sp. colony in a fringing reef.

We studied three coral species that persist in both inland bay and fringing reef environments in Curaçao: *Siderastrea siderea* (*n* = 80), *Siderastrea radians* (*n* = 36), and branching *Porites* sp. (*n* = 24) (Figure A1; Table S3). These corals have different life history strategies and morphologies: *S. siderea* is a massive, stress‐tolerant broadcast spawner, *S. radians* is an encrusting, weedy, brooding species, and branching *Porites* sp. is a branching, weedy, brooding species (Darling et al. 2012). In this study, we collected corals of the same morphology that we believe to be *Porites porites* (Figure A1D,E). Here, we refer to these samples as branching *Porites* sp. as this is a species complex and the species identity can only be confirmed by genetic analyses. For more details, see Appendix A.

Coral colonies of all species were tagged at the two fringing reefs at ~5 m and two inland bays at ~1 m in March 2020 based on coral local abundance and to sample corals from relatively similar light regimes (Figure 1H). In inland bays, corals occur in highest abundance from ~0–3 m, constrained by light availability in the turbid environment, whereas on the reefs, well‐developed coral communities begin to occur on a plateau at ~5 m. At three time points (March 2020, November 2020, and November 2021), 5–10 colonies of each coral species were collected by SCUBA at each site, but due to some tags going missing, not all colonies could be sampled at each time point. *Siderastrea radians* were only collected from the bays, and branching *Porites* sp. were only collected at the Santa Martha habitats (Table S3). Reef *S. radians* were not collected due to their presence in very shallow sandy areas far from the reef and the environmental loggers. *Porites* sp. was only collected in Santa Martha because it was uncommon at the collection sites in Spaanse Water. Corals were transported back to the Caribbean Research and Management of Biodiversity foundation (CARMABI) research station submerged in individual bags with seawater where 1–0.5 cm^2^ of combined coral tissue and skeleton were sampled from each colony using a Dremel (DREMEL) or sharp scissors. Samples were immediately placed into 1.8 mL sterile Nalgene cryogenic vials (Thermo Scientific) containing ~1 mL of RNAlater (Michael et al. 2013) and stored at −80°C to −20°C until extraction.

### 2.3 DNA Extraction

For each sample, genomic DNA was extracted from ~0.5 cm^2^ of combined tissue and skeleton using the DNeasy PowerSoil Pro DNA Isolation Kit (QIAGEN) according to the manufacturer's guidelines. Fragments were removed from RNAlater with tweezers and placed directly into digest buffer, thus, the analyses here represent a focus on tissue‐associated microbial members. A bead beater (BenchmarkScientific) was utilized for physical lysis and 25 μL of Proteinase K (Invitrogen) and 20 μL freshly prepared lysozyme solution (200 μg mL^−1^) were added to the digest buffer, DNA concentrations were determined using a Nanodrop 2000 (ThermoFisher) and a subset of samples were visualized on a 1% agarose gel to confirm isolation of genomic DNA.

### 2.4 PCR Amplification and Dual‐Barcoding

To determine Symbiodiniaceae and bacterial community composition, PCR amplification and dual‐barcoding were performed. Samples were amplified and dual‐barcoded using the Ex Taq HS DNA Polymerase (Takara Biotechnology), and purified using the GeneJet Purification kit (Thermo Scientific). The internal transcribed spacer 2 (ITS2) gene was amplified to determine Symbiodiniaceae community composition. PCR was performed using the SYM_5.8S and SYM_VAR_REV primers, which were found to capture the highest diversity of the ITS2 primers (Hume et al. 2018). The V4‐V5 region of the 16S rRNA gene was amplified to determine bacterial community composition using the primers 515F and 806R with degen, adapter, and linker sequences attached (Apprill et al. 2015; Parada et al. 2016). Full primer sequences are in Table S2. ITS2 PCR conditions were as follows: 30 cycles of 95°C denaturation for 1 min, 59°C annealing for 40 s, 72°C extension for 1 min, and a final extension at 72°C for 10 min. 16S PCR reactions were performed as follows: 95°C initial denaturation for 3 min followed by 35 cycles of 95°C denaturation for 40 s, 64°C annealing for 2 min, 72°C extension for 1 min, and a final extension at 72°C for 10 min. All PCR samples for both ITS2 and 16S were purified using the GeneJet Purification kit (Thermo Scientific), and dual‐barcoded using a second PCR with the following conditions: 6 cycles of 95°C for 1 min, 59°C for 1 min, and 72°C for 1 min. Successful amplification of all initial 16S and ITS2 PCR, as well as all dual‐barcoded samples, were confirmed via visualization on a 1% agarose gel. Negative controls of autoclaved ultrapure Type 1 MilliQ water (Millipore Sigma) were included for all PCR reactions, and negative controls from all PCR reactions were pooled before barcoding. A subset of samples of each species, location, and time point were quantified using a Qubit Flex fluorometer (Invitrogen). Samples were pooled into ITS2 and 16S libraries based on both visual band brightness and concentration. Pools were run on a 2% agarose gel stained with SYBR Green (Invitrogen), and visualized using a SmartBlue light transilluminator (Accuris) to prevent any UV damage to the DNA. Gel bands of the correct length were excised using a sterile razor blade and tweezers. Pools were then extracted and concentrated using the GeneJet Purification kit (Thermo Scientific). ITS2 and 16S libraries were pooled in a 1:2 ratio (ITS2:16S) and were sequenced at the Tufts Genomic Center on two lanes of Illumina MiSeq (paired‐end 2 × 250 bp).

### 2.5 Bioinformatics and Initial Data Processing

All analyses were performed using linux in the University of North Carolina computing cluster, and R in RStudio (Posit team 2023; R Core Team 2023). All samples were checked for adapter sequences (none were found), the first 4 bases created by degenerate primers were removed, and 16S and ITS2 reads were separated (*bbmap*) (Bushnell 2014). ITS2 sequences were submitted to SymPortal (Hume et al. 2019) for identification of ITS2 type profiles. Primer sequences were removed from 16S reads (*cutadapt*) (Martin 2011) and 16S data were processed following the *DADA2* pipeline (Callahan et al. 2016) to filter, trim, dereplicate, learn sequence error rates, merge paired reads, infer sequences, and remove chimeras, inferring 30,605 amplicon sequence variants (ASVs). 12 samples with low coverage failed quality assessment through *DADA2* and were removed. This included most (10) *Porites* sp. samples from November 2020. Read statistics can be found in Table S4. Taxonomy was assigned for each ASV using the Silva v. 138 database (Quast et al. 2013), *DECIPHER* (Wright 2023), the National Center for Biotechnology Information's nucleotide database using BLAST (Altschul et al. 1990) and TaxonKit (Shen and Ren 2021). A total of 1101 ASVs assigned to mitochondrial, chloroplast, and non‐bacterial members were removed, as well as 4 ASVs found in negative controls using *decontam* (Callahan et al. 2023). Lastly, samples with less than 1000 reads were removed from the dataset. This resulted in a final dataset of 27,653 ASVs and 123 samples, unrarefied and untrimmed (Hong et al. 2022; McMurdie and Holmes 2014; Willis 2019).

To ensure that the patterns we identified were consistent and robust, we compared results between full, trimmed, and rarefied datasets. Underrepresented ASVs were trimmed using *MCMC.OTU*, with a *z*‐score cutoff of −2.5 (removed one sample), ASV frequency cutoff of 0.0001 (resulted in 1006 ASVs), and ASV prevalence of 0.02, resulting in a trimmed dataset of 835 ASVs (Matz 2016). Rarefaction curves all reached asymptotes, and rarefaction was performed using *rrarefy* from *vegan* to 10,000 reads, ensuring that asymptotes were reached for all samples. Analyses of trimmed and rarefied datasets yielded negligible differences, and full datasets (untrimmed and unrarefied) were utilized for all further analyses.

For all data, comparisons were made between coral species (three levels, *S. siderea* , *S. radians* , and branching *Porites* sp.), as well as within‐species comparisons of habitats (two levels, bay vs. reef), location (two levels, Santa Martha vs. Spaanse Water), sites (four levels, Santa Martha Bay, Santa Martha Reef, Spaanse Water Bay, Spaanse Water Reef), and timepoints (three levels, March 2020, November 2020, November 2021).

### 2.6 Bacterial and Symbiodiniaceae Community Composition

Algal symbiont and bacterial community composition were visualized using relative abundance stacked bar plots across all location and habitat combinations using *ggplot2* (Wickham 2016). Symbiodiniaceae defining intragenomic variants (DIVs) were assessed at the genus and majority ITS2 type level to assess overarching trends of the symbiont community. Negative control DIVs were removed, and two samples with no DIVs were removed from the analysis. No diversity metrics were calculated for Symbiodiniaceae communities because of differences in ITS2 gene copy number between different ITS2 types, making diversity analyses inadvisable (Davies et al. 2023; Saad et al. 2020). Due to this, we assessed differences in the proportion of dominant majority ITS2 types between variables using multinomial models (*multinom* in *nnet*). Here, the dominant majority ITS2 type for a given sample is defined by ≥ 70% of a given ITS2 type (*sensu* Aichelman et al. (2025) with a more stringent cutoff). Specifically, models tested differences between coral species, habitats, sites, and timepoints and we utilized *anova* and *AIC* in *stats* for model selection (Table S5). We assessed model significance and effect sizes using *Anova* in *car* and performed post hoc comparisons using estimated marginal means (*emmeans*) with Tukey‐adjusted *p*‐values for multiple comparisons. Summaries of the proportion of dominant majority ITS2 types and selected comparisons can be found in Table S5.

Bacterial community composition was visualized at both the phylum and genus level, with bacteria less than 10% of each respective taxonomic level across the dataset grouped as “Other”. We also assessed differential abundance of taxa between reef habitats and locations for each coral species (*ALDEx2*; *microbiomeMarker*) (Cao et al. 2022; Fernandes et al. 2014). *ALDEx2* is known to be one of the most robust methods for differential abundance of relative abundance microbial data (Nearing et al. 2022).

### 2.7 Bacterial Diversity Analysis

Alpha diversity analyses were done on raw data to capture the full potential diversity of the sample, including rare members (McMurdie and Holmes 2013). Species Richness (observed ASVs), Evenness, Shannon's Index, Inverse Simpson's Index (*phyloseq*) and Phylogenetic diversity (Faith's D) (*phanghorn*, *DECIPHER*) were calculated for all coral species using the *estimate_richness* function from *phyloseq* (Knight et al. 2018; McMurdie and Holmes 2013; Schliep et al. 2023; Wright 2023). Generalized linear models (GLMs) were used to compare the effects of habitat (Bay vs. Reef), location (Spaanse Water vs. Santa Martha) and time point (March 2020, November 2020, and November 2021) with a random effect of genotype nested within each site for each coral species. Models with the smallest AICc (*MuMIn*) were selected for use, and model fit was assessed using DHARMa (Hartig 2024; Kamil 2022). Post hoc comparisons were assessed using estimated marginal means with Tukey comparison *p*‐value adjustments (*emmeans*) for all alpha diversity indices (Lenth et al. 2024). The selected models and additional information can be found in Table S6.

For bacterial beta diversity, beta dispersion (*betadisper*) was examined to determine dispersion while one‐way and pairwise PERMANOVAs (*adonis2* and *pairwiseadonis2*) were used to assess differences between Bray‐Curtis distances (Kandlikar and Cowen 2018; Oksanen et al. 2022). We tested for the effect of coral species, as well as within‐species effects of habitat ( *S. siderea* and branching *Porites* sp.), location ( *S. radians* ), timepoint (all species), site ( *S. siderea* ), habitat:timepoint ( *S. siderea* and branching *Porites* sp.), location:timepoint ( *S. radians* ), and site:timepoint ( *S. siderea* ) on Bray–Curtis distances. Principle Coordinate Analysis (PCoA) ordination plots were used to visualize beta diversity at site and timepoint.

To evaluate if dominant Symbiodiniaceae type and site independently structured bacterial community composition, we performed partial distance‐based redundancy analysis (dbRDA). We fit two partial models of the effect of symbiont type constrained by site, and site constrained by symbiont type and symbiont type on Bray–Curtis distances (*capscale*). Models were evaluated using permutation‐based ANOVA (*anova.cca*, permutations = 999).

## 3 Results

### 3.1 Symbiodiniaceae Community and Diversity

**FIGURE 2:**
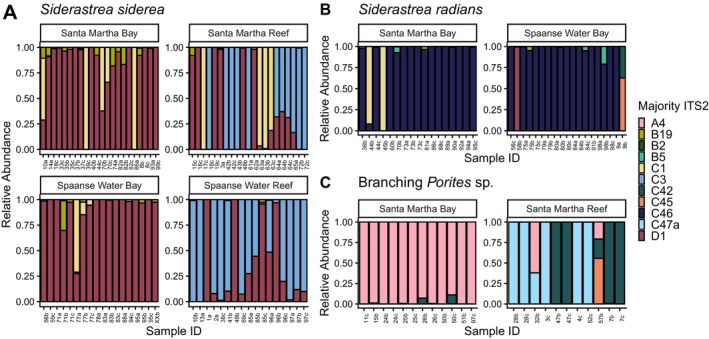
Relative abundance of majority ITS2 types at inland bays and fringing reefs for (A) *Siderastrea siderea*, (B) *Siderastrea radians*, and (C) branching *Porites* sp. Each color represents the majority ITS2 type. Bars represent individual samples which are labeled with the tag number and letters which represent timepoints (a = March 2020, b = November 2020, c = November 2021). Each site facet contains samples across all timepoints. For statistical comparisons of the proportion of samples dominated by these majority ITS2 types across coral species, sites and timepoints see Table S5.

Across all coral species, there were seven majority ITS2 types defined by “defining intragenomic variants” (DIVs) as assigned by SymPortal: *Symbiodinium* A4, *Cladocopium* C1, *Cladocopium* C3, *Cladocopium* C42, *Cladocopium* C46, *Cladocopium* C47a, and *Durusdinium* D1 (Table S5) (Hume et al. 2019). Proportions of coral samples dominated by a given ITS2 type (i.e., > 70% relative abundance) differed significantly between bays and reefs but not timepoint for *Siderastrea siderea* (*n* = 78) and branching *Porites* sp. (*n* = 23), and across timepoints for *Siderastrea radians* (*n* = 36) for a few ITS2 types (Figure 2, Table S5). C1 and D1 were found in both *S. siderea* and *S. radians* , while C3 was unique to *S. siderea* , and C46 was unique to *S. radians* . A4, C42, and C47a were all unique to branching *Porites* sp.

Significantly more bay *S. siderea* colonies (83%) were dominated by D1 compared to those on the reef (26%) (*p* = 0.0027). In contrast, more reef *S. siderea* (50%) were dominated by C3 compared to bay *S. siderea* (0%) (*p* = 0.0026) (Figure 2A, Table S5). There were no differences in the proportion of coral samples dominated by C1 across habitat and site comparisons, and no differences in D1, C3, or C1 across timepoints for *S. siderea* (Table S5).

Most *S. radians* colonies (89%) were dominated by C46 and had much smaller proportions of C1 (5.2%) and D1 (1.7%) dominated corals (Figure 2B, Table S5). There were no differences in the proportion of *S. radians* colonies dominated by any ITS2 types between Santa Martha Bay and Spaanse Water Bay and across timepoints (Table S5). Note that *S. radians* colonies were only sampled at both bay sites.

Branching *Porites* sp. colonies were dominated by A4 in Santa Martha Bay (100%), significantly more than Santa Martha Reef (0%) (*p* = < 0.001), and significantly more branching *Porites* sp. were dominated by C47a at Santa Martha Reef (46%) compared to Santa Martha Bay (0%) (*p* = 0.03) (Figure 2C, Table S5). There were no significant differences in branching *Porites* sp. colonies dominated by C42 across locations, and no differences in the proportion of A4, C42, and C47a dominated branching *Porites* sp. colonies over time (Table S5). Note that branching *Porites* sp. colonies were only sampled at Santa Martha Reef and Santa Martha Bay.

### 3.2 Bacterial Diversity

**FIGURE 3:**
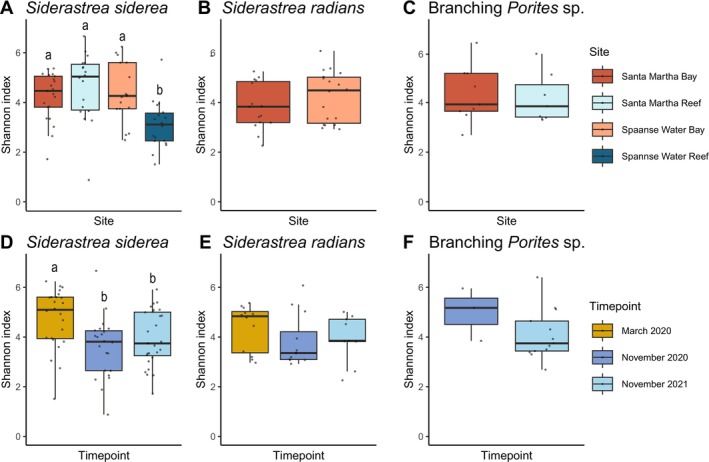
Shannon index of coral bacterial communities across sites (A–C) and timepoints (D–F) for *Siderastrea siderea* (A, D) *Siderastrea radians* (B, E), and branching *Porites* sp. (C, F). Letters indicate significant differences between sites and timepoints within each graph. Letters indicate significant differences between sites or timepoints within each graph (see Table S6). For all alpha diversity metrics and coral species comparisons, see Table S6 and Figure A2.

Alpha diversity was similar between coral species (Table S6). *Siderastrea siderea* (*n* = 74) displayed significantly higher Shannon Index and Richness in March 2020 compared to November 2020 and November 2021, significantly higher Inverse Simpson's Index in March 2020 compared to November 2020, and significantly higher Phylogenetic Diversity (Faith's D) in March 2020 and November 2021 compared to November 2020 (Figure 3; Table S6). *Siderastrea siderea* also displayed significantly lower Shannon Index and Richness at Spaanse Water Reef compared to all other sites (Figure 3 A,D; Table S6). Inverse Simpson's index was only significantly lower for *S. siderea* at Spaanse Water Reef compared to Santa Martha Reef, and Phylogenetic Diversity (Faith's *D*) was significantly lower at Spaanse Water Reef when compared to Santa Martha Bay and Santa Martha Reef (Table S6). Evenness remained the same for *S. siderea* across timepoints, habitat, location, and site comparisons (Table S6). All *S. radians* (*n* = 33) alpha diversity metrics except Phylogenetic Diversity (Faith's *D*) remained the same across all comparisons (Figure 3; Table S6). Phylogenetic Diversity (Faith's *D*) for *S. radians* was significantly different across timepoints, where November 2020 was significantly lower than March 2020 and November 2021 (Table S6). *Porites* sp. (*n* = 16) Richness and Phylogenetic Diversity (Faith's *D*) were significantly lower in November 2021 compared to November 2020; however, these results are likely influenced by small sample size for *Porites* sp. in November 2020 (*n* = 3). We found no significant differences between habitats for any alpha diversity metrics for *Porites* sp. (Figure 3; Table S6).

**FIGURE 4:**
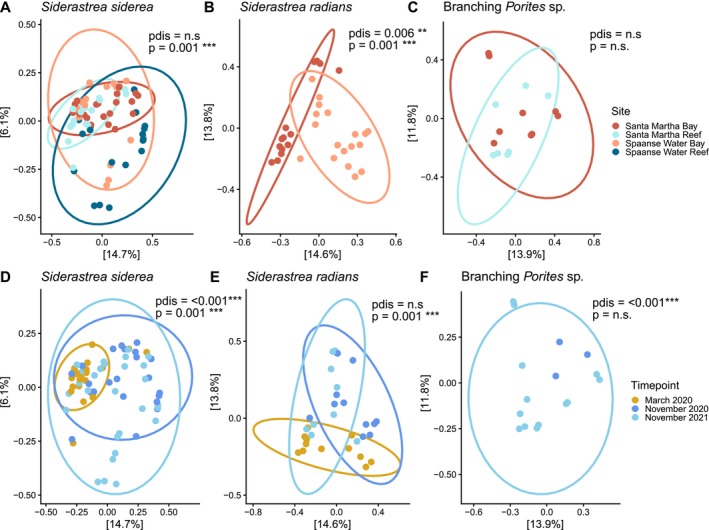
Beta diversity of coral bacterial communities across sites and timepoints for *Siderastrea siderea* (A, D) *Siderastrea radians* (B, E), and branching *Porites* sp. (C, F). Letters indicate significant differences between sites and timepoints. Differences in diversity between samples are visualized using Bray–Curtis distances in a Principal Coordinate Analysis (PCoA), where ellipses represent 95% confidence intervals and *p*‐values indicate significance levels comparing multivariate location, while *p*_dis_‐values compare dispersion (n.s. = not significant). See Table S7 for all statistical comparisons.

**FIGURE A2:**
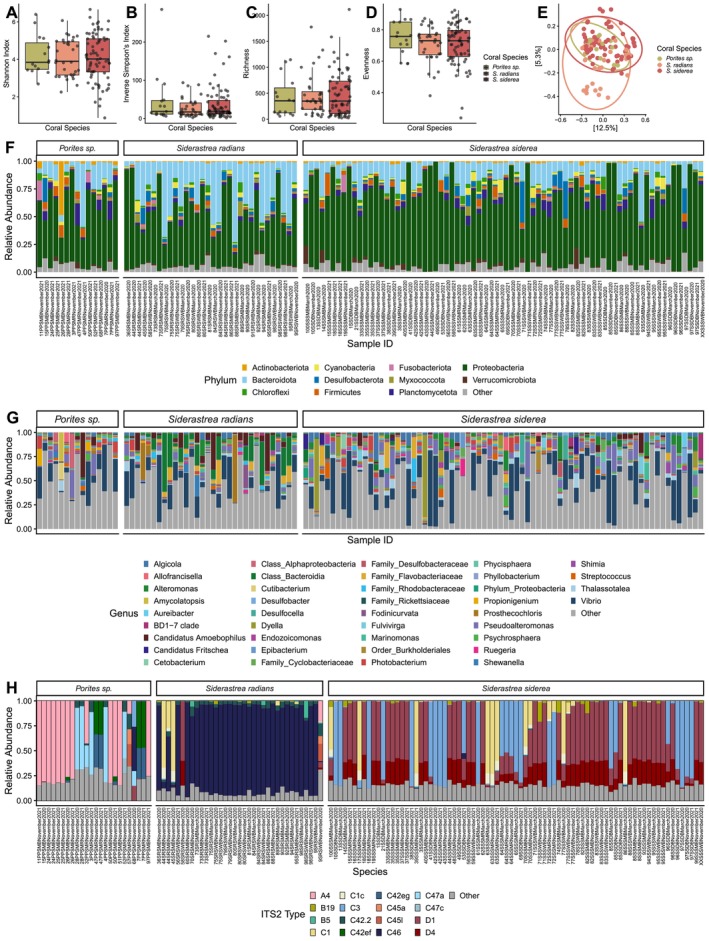
Coral species comparisons. (A–D) Alpha diversity indices comparisons of the bacterial community between coral species. (E) Beta diversity of the bacterial community between coral species. Differences in diversity between samples are visualized using Bray‐Curtis distances in a Principal Coordinate Analysis (PCoA), where ellipses represent 95% confidence intervals and *p*‐values indicate significance levels comparing multivariate location, while *p*_dis_‐values compare dispersion (n.s. = not significant). For comparisons of alpha diversity and beta diversity between coral species see Tables S6 and S7. (F) Relative abundance of bacterial community composition of all coral species. Colors represent bacterial phyla and (G) bacterial genera. Samples are labeled with full sample IDs which correspond vertically between panels F and G, and contain samples across sites and timepoints. (H) Relative abundance of the symbiont community (ITS2 types) of all coral species. Each bar represents an individual coral sample, and color and shade represent a ITS2 type, and colors are grouped by ITS2 clade. ITS2 types with less than 10% relative abundance are grouped and shown here in gray as “Other”. For statistical comparisons of the proportion of samples dominated by majority ITS2 types between coral species see Table S5.

Bacterial beta diversity differed significantly between all coral species (Figure A2; Table S7). For *S. siderea* , beta diversity was significantly different across habitat, timepoint, site, habitat:timepoint, and site:timepoint comparisons (Figure 4A,D; Table S7). Pairwise comparisons for all main effects and interactive effects were nearly all significantly different from each other (Table S7). *Siderastrea radians* beta diversity was significantly different across location, timepoint, and location:timepoint comparisons (Figure 4B,E; Table S7). Additionally, pairwise comparisons for location, timepoint, and location:timepoint interactions were virtually all significantly different from each other (Table S7). No significant differences in beta diversity were found for *Porites* sp. across habitat, timepoint, or habitat:timepoint comparisons (Figure 4C,F; Table S7).

After controlling for site, dominant Symbiodiniaceae type did not explain additional variation in bacterial community composition for *S. siderea* (*F* = 1.065, *p* = 0.305) or *S. radians* (*F* = 1.083, *p* = 0.253) using dbRDA, whereas site remained a significant predictor for both *S. siderea* (*F* = 1.705, *p* = 0.001) and *S. radians* (*F* = 2.333, *p* = 0.001) after controlling for dominant symbiont type. Using dbRDA was not possible for branching *Porites* sp. due to almost complete covariance of site and symbiont type.

### 3.3 Bacterial Community Composition

**FIGURE 5:**
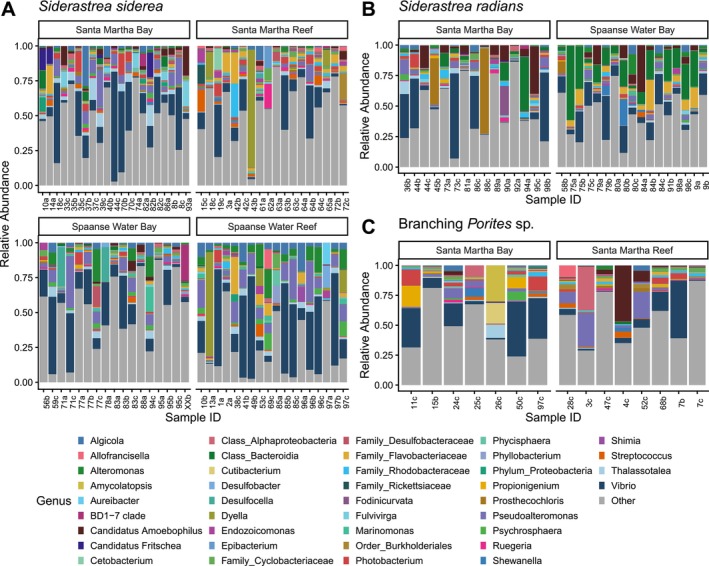
Relative abundance of bacterial community composition of (A) *Siderastrea siderea*, (B) *Siderastrea radians*, and (C) Branching *Porites* sp. at fringing reef and inland bay locations. Colors represent genera, and genera with lower than 10% relative abundance across the dataset are grouped into “Other”. Bars represent individual coral samples which are labeled with the tag number and letters which represent timepoints (a = March 2020, b = November 2020, c = November 2021). Each facet contains samples across all timepoints.

The community composition of bacteria revealed broad trends at the phylum level. Across species, locations, and habitats, the most abundant phyla were Proteobacteria and Bacteroidota (Figure A2 F).*Vibrio* was the most common bacterial genus in the bacterial communities across coral species, locations, and habitats based on ASV abundance (Figure 5, dark blue). Additionally, substantial variability was observed among individual colonies, shown primarily by differences in the relative abundance of genera including Alteromonas, Rhodobacteraceae, and Flavobacteriaceae (Figure 5). Species‐ and site‐specific differences include taxa such as *Pseudoalteromonas* (light purple) for *S. siderea* and *Alteromonas* (green) for *S. radians* (Figure 5). Branching *Porites* sp. showed lower variability in comparison to both *Siderastrea* species (Figure 5).

Analysis of differentially abundant bacteria revealed differences between timepoints and habitats, as well as interactive site, habitat:timepoint, and site:timepoint comparisons for *S. siderea* (Table S8). Timepoint differences were driven mainly by bacteria enriched in March 2020, such as members of Bacteriodota and *Phycisphaera*. Habitat differences were highlighted by enrichment of *Endozoicomonas* in reefs compared to bays (Table S8). For *S. siderea* , a host of bacteria were differentially abundant between sites such as *Thiohalophilus* in Spaanse Water Bay, and *Alteromonas* in Spaanse Water Reef (Table S8). Additionally, habitat:timepoint and site:timepoint comparisons revealed many differentially abundant bacteria for *S. siderea* (Table S8). *S. radians* displayed differentially abundant bacteria between timepoint, location, and location:timepoint differences (Table S8). These mainly included enriched bacteria in Spaanse Water Bay in March 2020 and November 2021. In Spaanse Water Bay in March 2020, there were differentially abundant such as *Thiohalophilus* that was also enriched in Spaanse Water Bay for *S. siderea* . No differentially abundant bacteria were found across habitat, timepoint, or any interactive comparisons for branching *Porites* sp. bacterial communities.

## 4 Discussion

### 4.1 Stress‐Resistant Algal Symbionts Facilitate Coral Survival in Extreme Inland Bay Habitats, But Do Not Fluctuate Across Seasons

Reef corals hosted significantly more generalist and stress‐sensitive symbionts including C3, a known heat sensitive symbiont (Fisher et al. 2012) for *S. siderea* , and less tolerant C47a (Grottoli et al. 2014) for branching *Porites* sp. (Figure 2). In the bays, corals hosted significantly stress tolerant Symbiodiniaceae including D1 for *S. siderea* and A4 for branching *Porites* sp. (Figure 2). D1 is a known thermotolerant symbiont correlated with coral stress tolerance and frequent occurrence in extreme and variable environments (Berkelmans and van Oppen 2006; Ros et al. 2021; Tanvet et al. 2023). Furthermore, Berkelmans and van Oppen (2006) found that corals that shuffled to *Durusdinium* from *Cladocopium* increased their bleaching tolerance by up to 1.5°C. Although D1 is thermotolerant, it still dominated 26% of reef *S. siderea* (Figure 2A; Table S5). This finding is corroborated by other studies that found high D1 abundance in more thermally stable environments (Baumann et al. 2018). One potential explanation for this trend is that *Durusdinium* sp. are increasing in abundance across environments in Caribbean corals due to their competitive dominance during heat stress events (Leveque et al. 2025). A4 is another known stress‐tolerant, shallow water specialist symbiont, likely influencing its success in the shallow, variable inland bays (LaJeunesse 2002). Furthermore, Grottoli et al. (2014) found that branching *P. divaricata* shuffled from C47 to A4 after repeated bleaching, likely facilitating their acclimatization under stress.

While we found site‐specific differences, Symbiodiniaceae communities of *S. siderea* and branching *Porites* sp. remained stable across seasonal timepoints (Table S5). This was interesting given that environmental conditions differ substantially between the dry and wet season, with wet seasons maintaining warmer temperatures including a heat stress event in fall 2020 (15.1 DHW), lower light levels, and generally more variable conditions (Figure 1D–H). Although some seasonal variation in Symbiodiniaceae communities has been documented (Ulstrup et al. 2008) long term (multi‐year) stability in Symbiodiniaceae appears to be more common, including in *S. siderea* and branching *P. furcata* (Cunning et al. 2024; Thornhill et al. 2006). We posit that here, *S. siderea* and branching *Porites* sp. are acclimatized to these seasonal environmental variations, which do not exceed their given stress thresholds for symbiont shuffling, and thus maintain stable Symbiodiniaceae communities over time.

*S. radians* did not show significant differences in their symbiont communities between the two bays (Figure 2B; Table S5). *S. radians* were dominated almost exclusively by C46—a unique association that is, to our knowledge, only found in other populations of *S. radians* and the closely related *S. stellata* (García et al. 2017). C46 is potentially adapted to *S. radians* ' life history as a small, encrusting, and weedy species that prefers shallow, lower light waters (Cunning et al. 2017; Darling et al. 2012; Monteiro et al. 2013). Although *S. radians* is able to associate with multiple symbionts, this symbiosis may be largely restricted to a majority of C46 by environmental filtering in the bays (Figure 2B) (Cunning et al. 2017; Monteiro et al. 2013). Indeed, Symbiodiniaceae associations across coral species are often constrained by light and depth (Bongaerts et al. 2013; Frade et al. 2008). Mean light levels (PAR) at the bay sites were similar to but generally lower than the reef sites, especially Spaanse Water Bay, despite the bay sites being shallower (Figure 1H). These lower light levels potentially drive the observed prevalence of C46 for *S. radians* , as well as A4 for branching *Porites* sp. (Figure 2). To our knowledge, the stress tolerance of C46 symbionts has not been tested, but we posit that they are tolerant to variable, multi‐stressor conditions due to their ubiquitous presence in bay *S. radians* , but reef *S. radians* samples and stress‐tolerance tests are needed to address this claim. Extreme and fluctuating abiotic conditions in the inland bays, especially more variable and higher temperatures, may filter Symbiodiniaceae availability to the coral (Figure 1E–H) (de Jong et al. 2025). Thus, identifying the Symbiodiniaceae communities present in the water and sediment that might be taken up by coral is also key to understanding differences in these communities between habitats and seasons.

### 4.2 Diverse Bacterial Associations Are Key to *Siderastrea* But Not Branching *Porites* sp. Success Across Environments, Seasons, and Timepoints

*Siderastrea* and branching *Porites* bacterial communities have been found to be highly diverse and variable across environmental gradients of depth, temperature, nutrients, and dissolved oxygen (Bonthond et al. 2018; Chu and Vollmer 2016; Speare et al. 2020). Here, we highlight differences in *S. siderea* and *S. radians* but not branching *Porites* sp. bacterial communities, diversity, and differential abundance across most variables, including habitats, locations, timepoints, and their interactions (Figures 3, 4, 5; Tables S6–S8). For example, *S. siderea* showed significant enrichment of *Endozoicomonas* in reef habitats compared to bays (Table S8). *Endozoicomonas* is a key core member of coral microbiomes that is potentially indicative of a stable, healthy microbiome, possibly reflecting the more stable environmental conditions in the reefs (Figure 1E–H) (Neave et al. 2017; Pogoreutz et al. 2022). *Siderastrea siderea* and *S. radians* bacterial communities even differed between the two bays, potentially due to differences in inorganic nutrients, industrial pollution, and ecotoxicological risk potential between bays (see de Jong et al. 2025). These nutrient levels correlate with differential abundance of bacteria with potential sulfur‐cycling capabilities in Spaanse Water Bay, such as *Thiotrichaceae* and *Thiohalophilus* for both *S. siderea* and *S. radians* (Table S8) (Li et al. 2022; Sorokin et al. 2007). Similar to these findings, other studies have shown that coral microbial communities are critical for holobiont nutrient cycling, and are diverse across strong environmental gradients related to water quality, urbanization, and CO_2_ seeps (Gantt et al. 2024; Messer et al. 2024; Morrow et al. 2015). Thus, site‐specific environmental conditions and water quality regimes are likely important for structuring *Siderastrea* bacterial communities, and microbial flexibility is potentially key for their success across environments (Table S8) (Camp et al. 2020; Voolstra and Ziegler 2020). Branching *Porites* sp. however showed no differences in bacterial communities across sites and time, potentially indicating that here, the branching *Porites* sp., coral host may play a larger role than habitat‐specific environmental factors in shaping their bacterial community via mechanisms such as nutrient cycling control, as seen in other coral species (Voolstra et al. 2024).

In contrast to Symbiodiniaceae, bacterial beta diversity and community composition of *S. siderea* and *S. radians* showed distinct shifts over time, especially between the warm, wet (November) and the cooler, dry (March) seasons (Figures 4 and 5). Previous studies have also found significant changes in coral bacterial communities over time and between seasons (Kimes et al. 2013), however there is very little information on these seasonal changes for *S. siderea* , *S. radians* , and branching *Porites* sp. Additionally, seasonal effects are hard to discern given that microbial communities can vary greatly from year to year and long‐term studies are therefore needed to separate seasonal from year‐to‐year variability (Epstein et al. 2019; Yang et al. 2017). Additionally, Vibrionaceae were more abundant during both wet seasons. Vibrionaceae are core coral microbiome members, where some species have important sulfur and nitrogen cycling capabilities, while other species have been implicated as coral pathogens (McCauley et al. 2023; Voolstra et al. 2024). *Alteromonas* and *Algicola*, bacterial genera which have been shown to be involved in coral holobiont nitrogen cycling, were also enriched during the wet season in 2021 for *S. siderea* and *S. radians* (Table S8) (Ceh et al. 2013; Nguyen et al. 2023). High abundance of these taxa is potentially a product of higher inorganic nutrient levels and temperatures during the wet season compared to the dry season (Figure 1E) (de Jong et al. 2025; Zaneveld et al. 2016). Overall, these findings underscore the importance of bacterial community flexibility of *Siderastrea* spp., plausibly providing these coral species with increased capacity for acclimatization (Voolstra and Ziegler 2020). This bacterial community divergence may augment coral survival in extreme environments and across seasons by facilitating diverse nutrient acquisition (Camp et al. 2020).

### 4.3 Coral Host Is a Significant Driver of Symbiodiniaceae and Bacterial Communities

Symbiodiniaceae community composition varied greatly between coral species, where each species hosted mainly unique majority ITS2 types (Figures 2, A2; Table S5). This work corroborates past findings that coral host is an important driver of Symbiodiniaceae community, including within *S. siderea* , *S. radians* , and branching *Porites* sp. (Baumann et al. 2018; Chu and Vollmer 2016; García et al. 2017). Many samples across coral species also contained multiple different Symbiodiniaceae species, acting as a potential adaptive advantage under variable, multiple‐stressor conditions as it allows corals to exist in a wider niche (Turnham et al. 2023). Coral are also known to have species‐specific microbial associations (Chu and Vollmer 2016) and our findings support this (Figure A2). Here, coral host species influenced the community composition and beta diversity of the coral bacterial communities, though not alpha diversity (Figure A2; Table S7). These species‐specific associations can be influenced by coral host morphology, biochemical composition, and nutrient cycling (Morrow et al. 2022; Voolstra et al. 2024). Recent work has also emphasized the importance of chemical signaling and nutrient transfer between bacteria and Symbiodiniaceae within the phycosphere, and the effect of micronutrients such as trace metals on these exchanges (Garrido et al. 2021; Reich et al. 2023). Furthermore, one study found that Symbiodiniaceae were a stronger driver of bacterial communities than environment (Yang et al. 2025). In contrast, we find that dominant Symbiodiniaceae type was not an important predictor of bacterial community composition, highlighting the potentially stronger role of the coral host in shaping the bacterial community of these corals. Overall, host species‐specificity of coral symbiont and microbiome communities suggests that each holobiont composition has unique nutritional linkages between hosts, symbionts, and bacteria.

It is important to address the potential for cryptic coral species that may be driving preferential symbiont and bacterial associations and resilience across environments (Grupstra, Gómez‐Corrales, et al. 2024). Cryptic lineages have been found in over 24 coral genera, including *Siderastrea* and branching *Porites*, which can be environmentally specialized (Aichelman et al. 2025; Forsman et al. 2020; Grupstra, Gómez‐Corrales, et al. 2024; Rippe et al. 2021). It is therefore possible that coral host cryptic lineage, rather than habitat, may drive differences in symbiont and bacterial community composition between bays and reefs. Especially in brooding species like *S. radians* and branching *Porites* sp., host‐symbiont‐microbiome coevolution may further affect holobiont success and resilience (Forsman et al. 2020; van Oppen and Medina 2020). Nevertheless, habitat and environmental conditions do play a key role, as these conditions also influence cryptic speciation, symbiont and bacterial availability, and coevolutionary relationships.

## 5 Conclusions

As the importance of the coral microbiome in facilitating holobiont resilience is increasingly recognized, environmentally extreme coral environments can serve as important natural laboratories to understand coral holobiont responses to future ocean conditions (Camp et al. 2018; Schoepf et al. 2023). Furthermore, coral microbiomes in extreme environments remain severely understudied in this regard (but see Camp et al. 2019; Ros et al. 2021; Tanvet et al. 2023), especially in the Caribbean. Here, we leverage the inland bays of Curaçao as a new natural laboratory (de Jong et al. 2025) and show that stress‐resistant Symbiodiniaceae and diverse bacteria with a wide variety of potential metabolisms likely play a key role in facilitating coral holobiont survival under highly fluctuating, multi‐stressor conditions. We further provide evidence for seasonal shifts in *Siderastrea* bacterial communities but emphasize the need for longer term studies across multiple years. Our work also has important implications for coral reef conservation. For example, the coral, Symbiodiniaceae and bacterial taxa that dominated in bay environments are potentially acclimatized to these extreme conditions, and thus may become more prevalent under future ocean conditions. Similarly, our work highlights the need to include extreme and marginal sites in coral conservation (Colton et al. 2022), since these sites represent potential coral resilience hotspots (Schoepf et al. 2023).

## Author Contributions

**Maya E. Powell:** formal analysis (lead), funding acquisition (equal), investigation (lead), visualization (lead), writing – original draft (lead), writing – review and editing (equal). **Sarah L. Solomon:** formal analysis (supporting), investigation (supporting), visualization (supporting), writing – review and editing (equal). **Verena Schoepf:** conceptualization (lead), funding acquisition (equal), investigation (supporting), project administration (equal), resources (equal), supervision (supporting), writing – review and editing (equal). **Karl D. Castillo:** funding acquisition (equal), project administration (equal), resources (equal), supervision (lead), writing – review and editing (equal).

## Funding

This research was made possible by grants from the International Coral Reef Society, Society for Integrative and Comparative Biology, Women Divers Hall of Fame, and the Phycological Society of America, along with the National Science Foundation Graduate Research Fellowship DGE‐2040435 to Maya Powell, National Science Foundation Grant OCE‐1459522 to Karl Castillo, and the University of Amsterdam MacGillavry Fellowship to Verena Schoepf.

## Conflicts of Interest

The authors declare no conflicts of interest.

## Supporting information


**Table S1:** Scheirer‐Ray‐Hare tests of daily averages of each environmental variable across habitats, timepoints, sites, habitat:timepoints, and site:timepoints. Levels are compared with post hoc Dunn tests with Benjamini‐Hochberg *p*‐value corrections for multiple comparisons (n.s., not significant). B, Bay; Mar., March; Nov., November; *R*, Reef; SM, Santa Martha Bay; SW, Spaanse Water.
**Table S2:** Primer sequences consisting of the forward or reverse adapter, linker, degen, and respective forward or reverse primer. The resulting products from PCR with these primers were prepared using Nextera Illumina barcodes (i5 and i7) and sequencing primers (P5 and P7) before sequencing.
**Table S3:** Number of samples collected for each species at each timepoint and site. Asterisks denote samples that were cut and transferred into appropriate volumes of RNA later upon returning to CARMABI (field station).
**Table S4:** Read counts throughout initial microbiome data processing through the *DADA2* pipeline.
**Table S5:** (A) Symbiodiniaceae community summary statistics examining the proportion of samples dominated by (> 70%) a majority ITS2 type. The proportion of samples dominated by majority ITS2 types is shown across coral species, and then within coral species comparisons of habitat or location, timepoint, and site (habitat: location interaction). (B) Multinomial model results of these comparisons of dominant majority ITS2 types and post hoc emmeans comparisons. n.s., not significant; SM, Santa Martha; SW, Spaanse Water.
**Table S6:** Alpha diversity metrics of bacterial communities compared across timepoint, habitat, location, and site (habitat: location) comparisons for all coral species, with a random effect of coral genotype using generalized linear models. The lowest AICc models were selected for use and are shown here. B, Bay; Mar., March; n.s., not significant; Nov., November; *R*, Reef; SM, Santa Martha; SW, Spaanse Water.
**Table S7:** Results from Pairwise Permutational Multivariate Analysis of Variance (PERMANOVA) of Bray–Curtis distances to assess differences in bacterial beta diversity between coral species and within coral species differences between habitats, timepoints, Sites, habitat:timepoints (or location:timepoint for *S. radians* ), and Site:timepoints. B, Bay; Mar., March; n.s., not significant; Nov., November; *R*, Reef; SM, Santa Martha; SW, Spaanse Water.
**Table S8:** Differential abundance of bacterial members using ALDEx2. Statistical comparisons of differentially abundant bacteria were made between coral species, and within‐species comparisons of timepoint, habitat, site, habitat:timepoint, and site:timepoint. Only significant differential abundance enrichments by GLM anova (ALDEx2) are shown (*p* < 0.05). Adjusted *p*‐values are Benjamini‐Hochberg adjusted for post hoc comparisons. B, Bay; Mar., March; Nov., November; *R*, Reef; SM, Santa Martha; SW, Spaanse Water.

## Data Availability

All ITS2 (Symbiodiniaceae) and 16S (bacterial) sequence data are archived under NCBI SRA number PRJNA1196340. All other data and associated code can be found on github at: https://github.com/mayapow/CW_2020 and in a corresponding Zenodo repository at: https://doi.org/10.5281/zenodo.20172748. Benefits Generated: Benefits from this research accrue from the sharing of our data and results on public databases as described above.

## Appendix A: Coral Species Comparisons

### Coral Species

*Siderastrea siderea* , *Siderastrea radians* , and branching *Porites* sp. all employ different life history strategies and all persist in both inland bay and fringing reef environments in Curaçao (Figure A1). The main coral life history strategies found in the Caribbean are stress‐tolerant, weedy, generalist, and competitive, which are characterized by morphology, growth and calcification, reproductive ecology, symbiont diversity, and more (Darling et al. 2012). *Siderastrea siderea* is a stress‐tolerant massive reef‐building coral that is abundant throughout the Caribbean (Figure A1A,B) (Darling et al. 2012). *S. siderea* utilizes a broadcast spawning reproductive strategy and is gonochoric (Baird et al. 2009). *S. radians* (Figure A1C) is an encrusting coral in the same *Siderastrea* genus that is a weedy, gonochoric, brooding species (Baird et al. 2009; Darling et al. 2012). *S. radians* is abundant in bays and reefs and is only found in very shallow areas (< 5 m) on fringing reefs and therefore was only sampled in bays for this study (Figure A1C) (de Jong et al. 2025; Vermeij et al. 2007). Caribbean branching *Porites* spp. (Figure A1D,E) make up a species complex, which includes *P. divaricata* , *P. furcata* , and *P. porites* , which are distinguished from each other by branch thickness (Prada et al. 2014; Tomascik and Sander 1987). Similar to *S. radians* , these branching *Porites* spp. also utilize a weedy life history strategy and are gonochoric and brooding species (Baird et al. 2009; Darling et al. 2012). Here, we collected *Porites porites* based on their morphology of thicker branches, light pink color, and “fluffy” polyps (Figure A1D,E). Further genetic analyses are needed to confirm the species identity of these *Porites* sp.

Corals were collected under Curaçao Ministry of Health, Environment & Nature permits to CARMABI: 2012/48584 in March 2020, and 2019/021824 in November 2020 and November 2021. Samples were transported under CITES II Permit 21CW021.
