# Supplementary material for: Stress‐Resistant Symbiodiniaceae and Diverse Bacterial Communities Promote Coral Persistence in Variable, Multi‐Stressor Environments
Source: Ecol Evol. 2026 Jun 2;16(6):e73783. doi: 10.1002/ece3.73783 (PMC13239945; doi:10.1002/ece3.73783)
Supplement: Supplementary file 1 — Table S1: Scheirer‐Ray‐Hare tests of daily averages of each environmental variable across habitats, timepoints, sites, habitat:timepoints, and site:timepoints. Levels are compared with post hoc Dunn tests with Benjamini‐Hochberg p‐value corrections for multiple comparisons (n.s., not significant). B, Bay; Mar., March; Nov., November; R, Reef; SM, Santa Martha Bay; SW, Spaanse Water. Table S2: Primer sequences consisting of the forward or reverse adapter, linker, degen, and respective forward or reverse primer. The resulting products from PCR with these primers were prepared using Nextera Illumina barcodes (i5 and i7) and sequencing primers (P5 and P7) before sequencing. Table S3: Number of samples collected for each species at each timepoint and site. Asterisks denote samples that were cut and transferred into appropriate volumes of RNA later upon returning to CARMABI (field station). Table S4: Read counts throughout initial microbiome data processing through the DADA2 pipeline. Table S5: (A) Symbiodiniaceae community summary statistics examining the proportion of samples dominated by (> 70%) a majority ITS2 type. The proportion of samples dominated by majority ITS2 types is shown across coral species, and then within coral species comparisons of habitat or location, timepoint, and site (habitat: location interaction). (B) Multinomial model results of these comparisons of dominant majority ITS2 types and post hoc emmeans comparisons. n.s., not significant; SM, Santa Martha; SW, Spaanse Water. Table S6: Alpha diversity metrics of bacterial communities compared across timepoint, habitat, location, and site (habitat: location) comparisons for all coral species, with a random effect of coral genotype using generalized linear models. The lowest AICc models were selected for use and are shown here. B, Bay; Mar., March; n.s., not significant; Nov., November; R, Reef; SM, Santa Martha; SW, Spaanse Water. Table S7: Results from Pairwise Permutational Multivariate Analysis of [file ECE3-16-e73783-s001.docx]

**Supplementary Information for:**

**Stress-resistant Symbiodiniaceae and diverse bacterial communities promote coral persistence in variable, multi-stressor environments**

Maya E. Powell^1*^, Sarah L. Solomon^2^, Verena Schoepf^2^, Karl D. Castillo^1,3^

^1^Environment, Ecology, and Energy Program, University of North Carolina at Chapel Hill, Chapel Hill, NC, USA

^2^Department of Freshwater and Marine Ecology, Institute for Biodiversity and Ecosystem Dynamics, University of Amsterdam, Amsterdam, The Netherlands

^3^Department of Earth, Marine and Environmental Sciences, University of North Carolina at Chapel Hill, Chapel Hill, NC, USA

^*^Corresponding author: [maya62928@gmail.com](mailto:maya62928@gmail.com)

**Supplementary Tables**

**Table S1.** Scheirer-Ray-Hare tests of daily averages of each environmental variable across habitats, timepoints, sites, habitat:timepoints, and site:timepoints. Levels are compared with post-hoc Dunn tests with Benjamini-Hochberg p-value corrections for multiple comparisons (n.s. = not significant). Mar. = March, Nov. = November. SM = Santa Martha Bay, SW = Spaanse Water. B = Bay, R = Reef.

| Daily Average Temperature (°C) | | | | | | | | |
| --- | --- | --- | --- | --- | --- | --- | --- | --- |
| **Effect** | **Comparison** | **df** | **Sum Sq** | **H** | **Z** | **p (un-adj)** | **p (adj)** | **sig. level** |
| Habitat | Bay - Reef | 1 | 132628 | 17.63 |  |  | <0.001 | *** |
| Timepoint |  | 2 | 1E+06 | 171 |  |  | <0.001 | *** |
|  | Mar. '20 - Nov. '20 |  |  |  | -12.665 | <0.001 | <0.001 | *** |
|  | Mar. '20 - Nov. '21 |  |  |  | -7.247 | <0.001 | <0.001 | *** |
|  | Nov. '20 - Nov. '21 |  |  |  | 6.916 | <0.001 | <0.001 | *** |
| Site |  | 3 | 162254 | 21.56 |  |  | <0.001 | *** |
|  | SMB - SMR |  |  |  | 2.823 | 0.005 | 0.014 | * |
|  | SMB - SWB |  |  |  | -1.565 | 0.117 | 0.141 | ns |
|  | SMR - SWB |  |  |  | -4.365 | <0.001 | <0.001 | *** |
|  | SMB - SWR |  |  |  | 1.068 | 0.286 | 0.286 | ns |
|  | SMR - SWR |  |  |  | -1.776 | 0.076 | 0.114 | ns |
|  | SWB - SWR |  |  |  | 2.636 | 0.008 | 0.017 | * |
| Habitat:Timepoint |  | 2 | 25048 | 3.329 |  |  | 0.189 | ns |
| Site:Timepoint |  | 6 | 50610 | 6.726 |  |  | 0.347 | ns |
| Daily Average pHT | | | | | | | | |
| **Effect** | **Comparison** | **df** | **Sum Sq** | **H** | **Z** | **p (un-adj)** | **p (adj)** | **sig. level** |
| Habitat | Bay - Reef | 1 | 60602 | 13.93 |  |  | <0.001 | *** |
| Timepoint |  | 2 | 278959 | 64.11 |  |  | <0.001 | *** |
|  | Mar. '20 - Nov. '20 |  |  |  | 1.657 | 0.097 | 0.097 | ns |
|  | Mar. '20 - Nov. '21 |  |  |  | 6.586 | <0.001 | <0.001 | *** |
|  | Nov. '20 - Nov. '21 |  |  |  | 7.395 | <0.001 | <0.001 | *** |
| Site |  | 3 | 249638 | 57.38 |  |  | <0.001 | *** |
|  | SMB - SMR |  |  |  | 0.458 | 0.647 | 0.971 | ns |
|  | SMB - SWB |  |  |  | 7.035 | <0.001 | <0.001 | *** |
|  | SMR - SWB |  |  |  | 6.402 | <0.001 | <0.001 | *** |
|  | SMB - SWR |  |  |  | 0.186 | 0.852 | 0.852 | ns |
|  | SMR - SWR |  |  |  | -0.245 | 0.806 | 0.968 | ns |
|  | SWB - SWR |  |  |  | -6.339 | <0.001 | <0.001 | *** |
| Habitat:Timepoint |  | 2 | 14675 | 3.373 |  |  | 0.185 | ns |
| Site:Timepoint |  | 6 | 95204 | 21.88 |  |  | 0.001 | ** |
|  | SMB Mar. '20 - SMB Nov. '20 |  |  |  | -0.614 | 0.539 | 0.726 | ns |
|  | SMB Mar. '20 - SMB Nov. '21 |  |  |  | 3.398 | 0.001 | 0.002 | ** |
|  | SMB Nov. '20 - SMB Nov. '21 |  |  |  | 5.856 | <0.001 | <0.001 | *** |
|  | SMB Mar. '20 - SMR Mar. '20 |  |  |  | 0.493 | 0.622 | 0.775 | ns |
|  | SMB Nov. '20 - SMR Mar. '20 |  |  |  | 1.235 | 0.217 | 0.333 | ns |
|  | SMB Nov. '21 - SMR Mar. '20 |  |  |  | -2.805 | 0.005 | 0.012 | * |
|  | SMB Mar. '20 - SMR Nov. '20 |  |  |  | 0.551 | 0.581 | 0.767 | ns |
|  | SMB Nov. '20 - SMR Nov. '20 |  |  |  | 1.819 | 0.069 | 0.117 | ns |
|  | SMB Nov. '21 - SMR Nov. '20 |  |  |  | -4.221 | <0.001 | <0.001 | *** |
|  | SMR Mar. '20 - SMR Nov. '20 |  |  |  | -0.070 | 0.944 | 0.974 | ns |
|  | SMB Mar. '20 - SMR Nov. '21 |  |  |  | 2.339 | 0.019 | 0.040 | * |
|  | SMB Nov. '20 - SMR Nov. '21 |  |  |  | 4.081 | <0.001 | <0.001 | *** |
|  | SMB Nov. '21 - SMR Nov. '21 |  |  |  | -1.202 | 0.230 | 0.344 | ns |
|  | SMR Mar. '20 - SMR Nov. '21 |  |  |  | 1.770 | 0.077 | 0.127 | ns |
|  | SMR Nov. '20 - SMR Nov. '21 |  |  |  | 2.580 | 0.010 | 0.022 | * |
|  | SMB Mar. '20 - SWB Mar. '20 |  |  |  | 0.176 | 0.860 | 0.931 | ns |
|  | SMB Nov. '20 - SWB Mar. '20 |  |  |  | 0.869 | 0.385 | 0.540 | ns |
|  | SMB Nov. '21 - SWB Mar. '20 |  |  |  | -3.328 | 0.001 | 0.003 | ** |
|  | SMR Mar. '20 - SWB Mar. '20 |  |  |  | -0.331 | 0.741 | 0.829 | ns |
|  | SMR Nov. '20 - SWB Mar. '20 |  |  |  | -0.351 | 0.725 | 0.826 | ns |
|  | SMR Nov. '21 - SWB Mar. '20 |  |  |  | -2.225 | 0.026 | 0.051 | ns |
|  | SMB Mar. '20 - SWB Nov. '20 |  |  |  | 4.276 | <0.001 | <0.001 | *** |
|  | SMB Nov. '20 - SWB Nov. '20 |  |  |  | 7.552 | <0.001 | <0.001 | *** |
|  | SMB Nov. '21 - SWB Nov. '20 |  |  |  | 1.033 | 0.302 | 0.433 | ns |
|  | SMR Mar. '20 - SWB Nov. '20 |  |  |  | 3.659 | <0.001 | 0.001 | *** |
|  | SMR Nov. '20 - SWB Nov. '20 |  |  |  | 5.763 | <0.001 | <0.001 | *** |
|  | SMR Nov. '21 - SWB Nov. '20 |  |  |  | 2.231 | 0.026 | 0.051 | ns |
|  | SWB Mar. '20 - SWB Nov. '20 |  |  |  | 4.250 | <0.001 | <0.001 | *** |
|  | SMB Mar. '20 - SWB Nov. '21 |  |  |  | 5.022 | <0.001 | <0.001 | *** |
|  | SMB Nov. '20 - SWB Nov. '21 |  |  |  | 8.706 | <0.001 | <0.001 | *** |
|  | SMB Nov. '21 - SWB Nov. '21 |  |  |  | 2.073 | 0.038 | 0.070 | ns |
|  | SMR Mar. '20 - SWB Nov. '21 |  |  |  | 4.405 | <0.001 | <0.001 | *** |
|  | SMR Nov. '20 - SWB Nov. '21 |  |  |  | 6.917 | <0.001 | <0.001 | *** |
|  | SMR Nov. '21 - SWB Nov. '21 |  |  |  | 3.187 | 0.001 | 0.004 | ** |
|  | SWB Mar. '20 - SWB Nov. '21 |  |  |  | 5.031 | <0.001 | <0.001 | *** |
|  | SWB Nov. '20 - SWB Nov. '21 |  |  |  | 1.135 | 0.257 | 0.376 | ns |
|  | SMB Mar. '20 - SWR Mar. '20 |  |  |  | 0.023 | 0.981 | 0.997 | ns |
|  | SMB Nov. '20 - SWR Mar. '20 |  |  |  | 0.533 | 0.594 | 0.769 | ns |
|  | SMB Nov. '21 - SWR Mar. '20 |  |  |  | -2.810 | 0.005 | 0.013 | * |
|  | SMR Mar. '20 - SWR Mar. '20 |  |  |  | -0.409 | 0.683 | 0.819 | ns |
|  | SMR Nov. '20 - SWR Mar. '20 |  |  |  | -0.426 | 0.670 | 0.819 | ns |
|  | SMR Nov. '21 - SWR Mar. '20 |  |  |  | -1.951 | 0.051 | 0.089 | ns |
|  | SWB Mar. '20 - SWR Mar. '20 |  |  |  | -0.130 | 0.897 | 0.939 | ns |
|  | SWB Nov. '20 - SWR Mar. '20 |  |  |  | -3.499 | <0.001 | 0.002 | ** |
|  | SWB Nov. '21 - SWR Mar. '20 |  |  |  | -4.114 | <0.001 | <0.001 | *** |
|  | SMB Mar. '20 - SWR Nov. '20 |  |  |  | 0.210 | 0.834 | 0.917 | ns |
|  | SMB Nov. '20 - SWR Nov. '20 |  |  |  | 1.246 | 0.213 | 0.334 | ns |
|  | SMB Nov. '21 - SWR Nov. '20 |  |  |  | -4.561 | <0.001 | <0.001 | *** |
|  | SMR Mar. '20 - SWR Nov. '20 |  |  |  | -0.402 | 0.688 | 0.796 | ns |
|  | SMR Nov. '20 - SWR Nov. '20 |  |  |  | -0.509 | 0.610 | 0.775 | ns |
|  | SMR Nov. '21 - SWR Nov. '20 |  |  |  | -2.942 | 0.003 | 0.009 | ** |
|  | SWB Mar. '20 - SWR Nov. '20 |  |  |  | -0.003 | 0.998 | 0.998 | ns |
|  | SWB Nov. '20 - SWR Nov. '20 |  |  |  | -6.069 | <0.001 | <0.001 | *** |
|  | SWB Nov. '21 - SWR Nov. '20 |  |  |  | -7.183 | <0.001 | <0.001 | *** |
|  | SWR Mar. '20 - SWR Nov. '20 |  |  |  | 0.146 | 0.884 | 0.941 | ns |
|  | SMB Mar. '20 - SWR Nov. '21 |  |  |  | 2.622 | 0.009 | 0.021 | * |
|  | SMB Nov. '20 - SWR Nov. '21 |  |  |  | 4.365 | <0.001 | <0.001 | *** |
|  | SMB Nov. '21 - SWR Nov. '21 |  |  |  | -0.723 | 0.470 | 0.646 | ns |
|  | SMR Mar. '20 - SWR Nov. '21 |  |  |  | 2.066 | 0.039 | 0.069 | ns |
|  | SMR Nov. '20 - SWR Nov. '21 |  |  |  | 2.930 | 0.003 | 0.009 | ** |
|  | SMR Nov. '21 - SWR Nov. '21 |  |  |  | 0.408 | 0.683 | 0.805 | ns |
|  | SWB Mar. '20 - SWR Nov. '21 |  |  |  | 2.520 | 0.012 | 0.025 | * |
|  | SWB Nov. '20 - SWR Nov. '21 |  |  |  | -1.676 | 0.094 | 0.151 | ns |
|  | SWB Nov. '21 - SWR Nov. '21 |  |  |  | -2.591 | 0.010 | 0.022 | * |
|  | SWR Mar. '20 - SWR Nov. '21 |  |  |  | 2.205 | 0.027 | 0.052 | ns |
|  | SWR Nov. '20 - SWR Nov. '21 |  |  |  | 3.272 | 0.001 | 0.003 | ** |
| Daily Average Dissolved Oxygen (mg/L) | | | | | | | | |
| **Effect** | **Comparison** | **df** | **Sum Sq** | **H** | **Z** | **p (un-adj)** | **p (adj)** | **sig. level** |
| Habitat | Bay - Reef | 1 | 331913 | 43.24 |  |  | <0.001 | *** |
| Timepoint |  | 2 | 274049 | 35.7 |  |  | <0.001 | *** |
|  | Mar. '20 - Nov. '20 |  |  |  | 3.007 | 0.003 | 0.004 | ** |
|  | Mar. '20 - Nov. '21 |  |  |  | -1.288 | 0.198 | 0.198 | ns |
|  | Nov. '20 - Nov. '21 |  |  |  | -5.609 | <0.001 | <0.001 | *** |
| Site |  | 3 | 635200 | 82.75 |  |  | <0.001 | *** |
|  | SMB - SMR |  |  |  | -1.144 | 0.253 | 0.303 | ns |
|  | SMB - SWB |  |  |  | 6.322 | <0.001 | <0.001 | *** |
|  | SMR - SWB |  |  |  | 7.182 | <0.001 | <0.001 | *** |
|  | SMB - SWR |  |  |  | -1.644 | 0.100 | 0.150 | ns |
|  | SMR - SWR |  |  |  | -0.445 | 0.656 | 0.656 | ns |
|  | SWB - SWR |  |  |  | -7.868 | <0.001 | <0.001 | *** |
| Habitat:Timepoint |  | 2 | 185440 | 24.16 |  |  | <0.001 | *** |
|  | Bay Mar. '20 - Bay Nov. '20 |  |  |  | 2.141 | 0.032 | 0.054 | ns |
|  | Bay Mar. '20 - Bay Nov. '21 |  |  |  | 1.314 | 0.189 | 0.236 | ns |
|  | Bay Nov. '20 - Bay Nov. '21 |  |  |  | -1.016 | 0.310 | 0.357 | ns |
|  | Bay Mar. '20 - Reef Mar. '20 |  |  |  | -1.358 | 0.174 | 0.238 | ns |
|  | Bay Nov. '20 - Reef Mar. '20 |  |  |  | -3.505 | <0.001 | 0.001 | ** |
|  | Bay Nov. '21 - Reef Mar. '20 |  |  |  | -2.738 | 0.006 | 0.013 | * |
|  | Bay Mar. '20 - Reef Nov. '20 |  |  |  | 0.799 | 0.424 | 0.454 | ns |
|  | Bay Nov. '20 - Reef Nov. '20 |  |  |  | -1.736 | 0.083 | 0.124 | ns |
|  | Bay Nov. '21 - Reef Nov. '20 |  |  |  | -0.674 | 0.500 | 0.500 | ns |
|  | Reef Mar. '20 - Reef Nov. '20 |  |  |  | 2.288 | 0.022 | 0.042 | * |
|  | Bay Mar. '20 - Reef Nov. '21 |  |  |  | -5.141 | <0.001 | <0.001 | *** |
|  | Bay Nov. '20 - Reef Nov. '21 |  |  |  | -9.091 | <0.001 | <0.001 | *** |
|  | Bay Nov. '21 - Reef Nov. '21 |  |  |  | -7.908 | <0.001 | <0.001 | *** |
|  | Reef Mar. '20 - Reef Nov. '21 |  |  |  | -3.175 | 0.001 | 0.004 | ** |
|  | Reef Nov. '20 - Reef Nov. '21 |  |  |  | -7.430 | <0.001 | <0.001 | *** |
| Site:Timepoint |  | 6 | 572279 | 74.55 |  |  | <0.001 | *** |
|  | SMB Mar. '20 - SMB Nov. '20 |  |  |  | -2.346 | 0.019 | 0.031 | * |
|  | SMB Mar. '20 - SMB Nov. '21 |  |  |  | -3.436 | 0.001 | 0.001 | ** |
|  | SMB Nov. '20 - SMB Nov. '21 |  |  |  | -1.407 | 0.159 | 0.202 | ns |
|  | SMB Mar. '20 - SMR Mar. '20 |  |  |  | -1.446 | 0.148 | 0.196 | ns |
|  | SMB Nov. '20 - SMR Mar. '20 |  |  |  | 0.574 | 0.566 | 0.612 | ns |
|  | SMB Nov. '21 - SMR Mar. '20 |  |  |  | 1.652 | 0.099 | 0.138 | ns |
|  | SMB Mar. '20 - SMR Nov. '20 |  |  |  | -1.194 | 0.233 | 0.274 | ns |
|  | SMB Nov. '20 - SMR Nov. '20 |  |  |  | 1.441 | 0.150 | 0.194 | ns |
|  | SMB Nov. '21 - SMR Nov. '20 |  |  |  | 2.802 | 0.005 | 0.009 | ** |
|  | SMR Mar. '20 - SMR Nov. '20 |  |  |  | 0.527 | 0.598 | 0.637 | ns |
|  | SMB Mar. '20 - SMR Nov. '21 |  |  |  | -6.199 | <0.001 | <0.001 | *** |
|  | SMB Nov. '20 - SMR Nov. '21 |  |  |  | -4.860 | <0.001 | <0.001 | *** |
|  | SMB Nov. '21 - SMR Nov. '21 |  |  |  | -3.546 | <0.001 | 0.001 | *** |
|  | SMR Mar. '20 - SMR Nov. '21 |  |  |  | -4.470 | <0.001 | <0.001 | *** |
|  | SMR Nov. '20 - SMR Nov. '21 |  |  |  | -6.080 | <0.001 | <0.001 | *** |
|  | SMB Mar. '20 - SWB Mar. '20 |  |  |  | -3.103 | 0.002 | 0.004 | ** |
|  | SMB Nov. '20 - SWB Mar. '20 |  |  |  | -1.371 | 0.170 | 0.212 | ns |
|  | SMB Nov. '21 - SWB Mar. '20 |  |  |  | -0.285 | 0.775 | 0.812 | ns |
|  | SMR Mar. '20 - SWB Mar. '20 |  |  |  | -1.634 | 0.102 | 0.141 | ns |
|  | SMR Nov. '20 - SWB Mar. '20 |  |  |  | -2.443 | 0.015 | 0.025 | * |
|  | SMR Nov. '21 - SWB Mar. '20 |  |  |  | 2.625 | 0.009 | 0.015 | * |
|  | SMB Mar. '20 - SWB Nov. '20 |  |  |  | 1.935 | 0.053 | 0.079 | ns |
|  | SMB Nov. '20 - SWB Nov. '20 |  |  |  | 5.406 | <0.001 | <0.001 | *** |
|  | SMB Nov. '21 - SWB Nov. '20 |  |  |  | 6.655 | <0.001 | <0.001 | *** |
|  | SMR Mar. '20 - SWB Nov. '20 |  |  |  | 3.526 | <0.001 | 0.001 | ** |
|  | SMR Nov. '20 - SWB Nov. '20 |  |  |  | 3.945 | <0.001 | <0.001 | *** |
|  | SMR Nov. '21 - SWB Nov. '20 |  |  |  | 9.468 | <0.001 | <0.001 | *** |
|  | SWB Mar. '20 - SWB Nov. '20 |  |  |  | 5.377 | <0.001 | <0.001 | *** |
|  | SMB Mar. '20 - SWB Nov. '21 |  |  |  | 1.842 | 0.066 | 0.096 | ns |
|  | SMB Nov. '20 - SWB Nov. '21 |  |  |  | 5.178 | <0.001 | <0.001 | *** |
|  | SMB Nov. '21 - SWB Nov. '21 |  |  |  | 6.405 | <0.001 | <0.001 | *** |
|  | SMR Mar. '20 - SWB Nov. '21 |  |  |  | 3.411 | 0.001 | 0.001 | ** |
|  | SMR Nov. '20 - SWB Nov. '21 |  |  |  | 3.759 | <0.001 | <0.001 | *** |
|  | SMR Nov. '21 - SWB Nov. '21 |  |  |  | 9.210 | <0.001 | <0.001 | *** |
|  | SWB Mar. '20 - SWB Nov. '21 |  |  |  | 5.237 | <0.001 | <0.001 | *** |
|  | SWB Nov. '20 - SWB Nov. '21 |  |  |  | -0.072 | 0.942 | 0.942 | ns |
|  | SMB Mar. '20 - SWR Mar. '20 |  |  |  | -3.598 | <0.001 | 0.001 | *** |
|  | SMB Nov. '20 - SWR Mar. '20 |  |  |  | -2.155 | 0.031 | 0.049 | * |
|  | SMB Nov. '21 - SWR Mar. '20 |  |  |  | -1.242 | 0.214 | 0.257 | ns |
|  | SMR Mar. '20 - SWR Mar. '20 |  |  |  | -2.321 | 0.020 | 0.033 | * |
|  | SMR Nov. '20 - SWR Mar. '20 |  |  |  | -3.044 | 0.002 | 0.005 | ** |
|  | SMR Nov. '21 - SWR Mar. '20 |  |  |  | 1.294 | 0.196 | 0.239 | ns |
|  | SWB Mar. '20 - SWR Mar. '20 |  |  |  | -0.889 | 0.374 | 0.418 | ns |
|  | SWB Nov. '20 - SWR Mar. '20 |  |  |  | -5.474 | <0.001 | <0.001 | *** |
|  | SWB Nov. '21 - SWR Mar. '20 |  |  |  | -5.368 | <0.001 | <0.001 | *** |
|  | SMB Mar. '20 - SWR Nov. '20 |  |  |  | -1.063 | 0.288 | 0.333 | ns |
|  | SMB Nov. '20 - SWR Nov. '20 |  |  |  | 1.604 | 0.109 | 0.146 | ns |
|  | SMB Nov. '21 - SWR Nov. '20 |  |  |  | 2.960 | 0.003 | 0.006 | ** |
|  | SMR Mar. '20 - SWR Nov. '20 |  |  |  | 0.651 | 0.515 | 0.566 | ns |
|  | SMR Nov. '20 - SWR Nov. '20 |  |  |  | 0.163 | 0.870 | 0.884 | ns |
|  | SMR Nov. '21 - SWR Nov. '20 |  |  |  | 6.219 | <0.001 | <0.001 | *** |
|  | SWB Mar. '20 - SWR Nov. '20 |  |  |  | 2.564 | 0.010 | 0.018 | * |
|  | SWB Nov. '20 - SWR Nov. '20 |  |  |  | -3.779 | <0.001 | <0.001 | *** |
|  | SWB Nov. '21 - SWR Nov. '20 |  |  |  | -3.598 | <0.001 | 0.001 | *** |
|  | SWR Mar. '20 - SWR Nov. '20 |  |  |  | 3.145 | 0.002 | 0.004 | ** |
|  | SMB Mar. '20 - SWR Nov. '21 |  |  |  | -4.874 | <0.001 | <0.001 | *** |
|  | SMB Nov. '20 - SWR Nov. '21 |  |  |  | -3.165 | 0.002 | 0.003 | ** |
|  | SMB Nov. '21 - SWR Nov. '21 |  |  |  | -1.668 | 0.095 | 0.137 | ns |
|  | SMR Mar. '20 - SWR Nov. '21 |  |  |  | -2.996 | 0.003 | 0.005 | ** |
|  | SMR Nov. '20 - SWR Nov. '21 |  |  |  | -4.595 | <0.001 | <0.001 | *** |
|  | SMR Nov. '21 - SWR Nov. '21 |  |  |  | 2.149 | 0.032 | 0.049 | * |
|  | SWB Mar. '20 - SWR Nov. '21 |  |  |  | -0.996 | 0.319 | 0.363 | ns |
|  | SWB Nov. '20 - SWR Nov. '21 |  |  |  | -8.573 | <0.001 | <0.001 | *** |
|  | SWB Nov. '21 - SWR Nov. '21 |  |  |  | -8.257 | <0.001 | <0.001 | *** |
|  | SWR Mar. '20 - SWR Nov. '21 |  |  |  | 0.186 | 0.852 | 0.879 | ns |
|  | SWR Nov. '20 - SWR Nov. '21 |  |  |  | -4.757 | <0.001 | <0.001 | *** |
| Daily Average Salinity (ppt) | | | | | | | | |
| **Effect** | **Comparison** | **df** | **Sum Sq** | **H** | **Z** | **p (un-adj)** | **p (adj)** | **sig. level** |
| Habitat | Bay - Reef | 1 | 217822 | 42.33 |  |  | <0.001 | *** |
| Timepoint |  | 2 | 127909 | 24.86 |  |  | <0.001 | *** |
|  | Mar. '20 - Nov. '20 |  |  |  | 3.522 | <0.001 | 0.001 | *** |
|  | Mar. '20 - Nov. '21 |  |  |  | 4.959 | <0.001 | <0.001 | *** |
|  | Nov. '20 - Nov. '21 |  |  |  | 1.024 | 0.306 | 0.306 | ns |
| Site |  | 3 | 506798 | 98.48 |  |  | <0.001 | *** |
|  | SMB - SMR |  |  |  | 2.982 | 0.003 | 0.003 | ** |
|  | SMB - SWB |  |  |  | -6.524 | <0.001 | <0.001 | *** |
|  | SMR - SWB |  |  |  | -9.224 | <0.001 | <0.001 | *** |
|  | SMB - SWR |  |  |  | -0.228 | 0.820 | 0.820 | ns |
|  | SMR - SWR |  |  |  | -3.110 | 0.002 | 0.003 | ** |
|  | SWB - SWR |  |  |  | 6.061 | <0.001 | <0.001 | *** |
| Habitat:Timepoint |  | 2 | 215602 | 41.9 |  |  | <0.001 | *** |
|  | Bay Mar. '20 - Bay Nov. '20 |  |  |  | 2.815 | 0.005 | 0.010 | * |
|  | Bay Mar. '20 - Bay Nov. '21 |  |  |  | -0.163 | 0.871 | 1.000 | ns |
|  | Bay Nov. '20 - Bay Nov. '21 |  |  |  | -3.538 | <0.001 | 0.001 | ** |
|  | Bay Mar. '20 - Reef Mar. '20 |  |  |  | -0.010 | 0.992 | 0.992 | ns |
|  | Bay Nov. '20 - Reef Mar. '20 |  |  |  | -2.679 | 0.007 | 0.014 | * |
|  | Bay Nov. '21 - Reef Mar. '20 |  |  |  | 0.141 | 0.888 | 0.951 | ns |
|  | Bay Mar. '20 - Reef Nov. '20 |  |  |  | 2.085 | 0.037 | 0.056 | ns |
|  | Bay Nov. '20 - Reef Nov. '20 |  |  |  | -0.257 | 0.798 | 0.997 | ns |
|  | Bay Nov. '21 - Reef Nov. '20 |  |  |  | 2.476 | 0.013 | 0.022 | * |
|  | Reef Mar. '20 - Reef Nov. '20 |  |  |  | 2.018 | 0.044 | 0.059 | ns |
|  | Bay Mar. '20 - Reef Nov. '21 |  |  |  | 7.317 | <0.001 | <0.001 | *** |
|  | Bay Nov. '20 - Reef Nov. '21 |  |  |  | 4.858 | <0.001 | <0.001 | *** |
|  | Bay Nov. '21 - Reef Nov. '21 |  |  |  | 9.174 | <0.001 | <0.001 | *** |
|  | Reef Mar. '20 - Reef Nov. '21 |  |  |  | 6.899 | <0.001 | <0.001 | *** |
|  | Reef Nov. '20 - Reef Nov. '21 |  |  |  | 4.032 | <0.001 | <0.001 | *** |
| Site:Timepoint |  | 6 | 390518 | 75.89 |  |  | <0.001 | *** |
|  | SMB Mar. '20 - SMB Nov. '20 |  |  |  | 3.874 | <0.001 | <0.001 | *** |
|  | SMB Mar. '20 - SMB Nov. '21 |  |  |  | 3.315 | 0.001 | 0.002 | ** |
|  | SMB Nov. '20 - SMB Nov. '21 |  |  |  | -0.918 | 0.358 | 0.473 | ns |
|  | SMB Mar. '20 - SMR Mar. '20 |  |  |  | -0.158 | 0.875 | 0.888 | ns |
|  | SMB Nov. '20 - SMR Mar. '20 |  |  |  | -3.894 | <0.001 | <0.001 | *** |
|  | SMB Nov. '21 - SMR Mar. '20 |  |  |  | -3.349 | 0.001 | 0.002 | ** |
|  | SMB Mar. '20 - SMR Nov. '20 |  |  |  | 4.150 | <0.001 | <0.001 | *** |
|  | SMB Nov. '20 - SMR Nov. '20 |  |  |  | 1.072 | 0.284 | 0.390 | ns |
|  | SMB Nov. '21 - SMR Nov. '20 |  |  |  | 1.827 | 0.068 | 0.099 | ns |
|  | SMR Mar. '20 - SMR Nov. '20 |  |  |  | 4.183 | <0.001 | <0.001 | *** |
|  | SMB Mar. '20 - SMR Nov. '21 |  |  |  | 6.471 | <0.001 | <0.001 | *** |
|  | SMB Nov. '20 - SMR Nov. '21 |  |  |  | 2.439 | 0.015 | 0.023 | * |
|  | SMB Nov. '21 - SMR Nov. '21 |  |  |  | 3.728 | <0.001 | <0.001 | *** |
|  | SMR Mar. '20 - SMR Nov. '21 |  |  |  | 6.367 | <0.001 | <0.001 | *** |
|  | SMR Nov. '20 - SMR Nov. '21 |  |  |  | 0.721 | 0.471 | 0.565 | ns |
|  | SMB Mar. '20 - SWB Mar. '20 |  |  |  | 0.423 | 0.672 | 0.752 | ns |
|  | SMB Nov. '20 - SWB Mar. '20 |  |  |  | -3.359 | 0.001 | 0.002 | ** |
|  | SMB Nov. '21 - SWB Mar. '20 |  |  |  | -2.768 | 0.006 | 0.010 | ** |
|  | SMR Mar. '20 - SWB Mar. '20 |  |  |  | 0.564 | 0.573 | 0.652 | ns |
|  | SMR Nov. '20 - SWB Mar. '20 |  |  |  | -3.741 | <0.001 | 0.001 | *** |
|  | SMR Nov. '21 - SWB Mar. '20 |  |  |  | -5.854 | <0.001 | <0.001 | *** |
|  | SMB Mar. '20 - SWB Nov. '20 |  |  |  | 0.861 | 0.389 | 0.485 | ns |
|  | SMB Nov. '20 - SWB Nov. '20 |  |  |  | -3.412 | 0.001 | 0.001 | ** |
|  | SMB Nov. '21 - SWB Nov. '20 |  |  |  | -2.772 | 0.006 | 0.010 | ** |
|  | SMR Mar. '20 - SWB Nov. '20 |  |  |  | 0.999 | 0.318 | 0.428 | ns |
|  | SMR Nov. '20 - SWB Nov. '20 |  |  |  | -3.739 | <0.001 | <0.001 | *** |
|  | SMR Nov. '21 - SWB Nov. '20 |  |  |  | -6.379 | <0.001 | <0.001 | *** |
|  | SWB Mar. '20 - SWB Nov. '20 |  |  |  | 0.379 | 0.705 | 0.775 | ns |
|  | SMB Mar. '20 - SWB Nov. '21 |  |  |  | -2.745 | 0.006 | 0.010 | * |
|  | SMB Nov. '20 - SWB Nov. '21 |  |  |  | -7.536 | <0.001 | <0.001 | *** |
|  | SMB Nov. '21 - SWB Nov. '21 |  |  |  | -7.341 | <0.001 | <0.001 | *** |
|  | SMR Mar. '20 - SWB Nov. '21 |  |  |  | -2.441 | 0.015 | 0.024 | * |
|  | SMR Nov. '20 - SWB Nov. '21 |  |  |  | -6.822 | <0.001 | <0.001 | *** |
|  | SMR Nov. '21 - SWB Nov. '21 |  |  |  | -11.256 | <0.001 | <0.001 | *** |
|  | SWB Mar. '20 - SWB Nov. '21 |  |  |  | -3.173 | 0.002 | 0.003 | ** |
|  | SWB Nov. '20 - SWB Nov. '21 |  |  |  | -4.194 | <0.001 | <0.001 | *** |
|  | SMB Mar. '20 - SWR Mar. '20 |  |  |  | 0.578 | 0.563 | 0.652 | ns |
|  | SMB Nov. '20 - SWR Mar. '20 |  |  |  | -2.954 | 0.003 | 0.006 | ** |
|  | SMB Nov. '21 - SWR Mar. '20 |  |  |  | -2.364 | 0.018 | 0.028 | * |
|  | SMR Mar. '20 - SWR Mar. '20 |  |  |  | 0.708 | 0.479 | 0.565 | ns |
|  | SMR Nov. '20 - SWR Mar. '20 |  |  |  | -3.421 | 0.001 | 0.001 | ** |
|  | SMR Nov. '21 - SWR Mar. '20 |  |  |  | -5.219 | <0.001 | <0.001 | *** |
|  | SWB Mar. '20 - SWR Mar. '20 |  |  |  | 0.178 | 0.859 | 0.886 | ns |
|  | SWB Nov. '20 - SWR Mar. '20 |  |  |  | -0.157 | 0.876 | 0.876 | ns |
|  | SWB Nov. '21 - SWR Mar. '20 |  |  |  | 3.135 | 0.002 | 0.003 | ** |
|  | SMB Mar. '20 - SWR Nov. '20 |  |  |  | -0.359 | 0.720 | 0.779 | ns |
|  | SMB Nov. '20 - SWR Nov. '20 |  |  |  | -3.933 | <0.001 | <0.001 | *** |
|  | SMB Nov. '21 - SWR Nov. '20 |  |  |  | -3.407 | 0.001 | 0.001 | ** |
|  | SMR Mar. '20 - SWR Nov. '20 |  |  |  | -0.202 | 0.840 | 0.880 | ns |
|  | SMR Nov. '20 - SWR Nov. '20 |  |  |  | -4.233 | <0.001 | <0.001 | *** |
|  | SMR Nov. '21 - SWR Nov. '20 |  |  |  | -6.266 | <0.001 | <0.001 | *** |
|  | SWB Mar. '20 - SWR Nov. '20 |  |  |  | -0.746 | 0.456 | 0.557 | ns |
|  | SWB Nov. '20 - SWR Nov. '20 |  |  |  | -1.175 | 0.240 | 0.337 | ns |
|  | SWB Nov. '21 - SWR Nov. '20 |  |  |  | 2.074 | 0.038 | 0.057 | ns |
|  | SWR Mar. '20 - SWR Nov. '20 |  |  |  | -0.876 | 0.381 | 0.483 | ns |
|  | SMB Mar. '20 - SWR Nov. '21 |  |  |  | 4.543 | <0.001 | <0.001 | *** |
|  | SMB Nov. '20 - SWR Nov. '21 |  |  |  | 0.299 | 0.765 | 0.814 | ns |
|  | SMB Nov. '21 - SWR Nov. '21 |  |  |  | 1.382 | 0.167 | 0.240 | ns |
|  | SMR Mar. '20 - SWR Nov. '21 |  |  |  | 4.521 | <0.001 | <0.001 | *** |
|  | SMR Nov. '20 - SWR Nov. '21 |  |  |  | -0.918 | 0.359 | 0.464 | ns |
|  | SMR Nov. '21 - SWR Nov. '21 |  |  |  | -2.454 | 0.014 | 0.023 | * |
|  | SWB Mar. '20 - SWR Nov. '21 |  |  |  | 3.957 | <0.001 | <0.001 | *** |
|  | SWB Nov. '20 - SWR Nov. '21 |  |  |  | 4.180 | <0.001 | <0.001 | *** |
|  | SWB Nov. '21 - SWR Nov. '21 |  |  |  | 9.019 | <0.001 | <0.001 | *** |
|  | SWR Mar. '20 - SWR Nov. '21 |  |  |  | 3.451 | 0.001 | 0.001 | ** |
|  | SWR Nov. '20 - SWR Nov. '21 |  |  |  | 4.512 | <0.001 | <0.001 | *** |
| Daily Average Photosynthetically Active Radiation (PAR) (umol/m2/s) | | | | | | | | |
| **Effect** | **Comparison** | **df** | **Sum Sq** | **H** | **Z** | **p (un-adj)** | **p (adj)** | **sig. level** |
| Habitat | Bay - Reef | 1 | 108578 | 26.08 |  |  | <0.001 | *** |
| Timepoint |  | 2 | 138935 | 33.38 |  |  | <0.001 | *** |
|  | Mar. '20 - Nov. '20 |  |  |  | 4.296 | <0.001 | <0.001 | *** |
|  | Mar. '20 - Nov. '21 |  |  |  | 5.979 | <0.001 | <0.001 | *** |
|  | Nov. '20 - Nov. '21 |  |  |  | 1.990 | 0.047 | 0.047 | * |
| Site |  | 3 | 208934 | 50.19 |  |  | <0.001 | *** |
|  | SMB - SMR |  |  |  | -1.878 | 0.060 | 0.091 | ns |
|  | SMB - SWB |  |  |  | 4.849 | <0.001 | <0.001 | *** |
|  | SMR - SWB |  |  |  | 5.592 | <0.001 | <0.001 | *** |
|  | SMB - SWR |  |  |  | -1.415 | 0.157 | 0.188 | ns |
|  | SMR - SWR |  |  |  | 0.711 | 0.477 | 0.477 | ns |
|  | SWB - SWR |  |  |  | -6.037 | <0.001 | <0.001 | *** |
| Habitat:Timepoint |  | 2 | 385 | 0.092 |  |  | 0.955 | ns |
| Site:Timepoint |  | 6 | 19373 | 4.654 |  |  | 0.589 | ns |

**Table S2.** Primer sequences consisting of the forward or reverse adapter, linker, degen, and respective forward or reverse primer. The resulting products from PCR with these primers were prepared using Nextera Illumina barcodes (i5 and i7) and sequencing primers (P5 and P7) before sequencing.

| **Primer** | Adapter *Linker,* **Degen,** Primer |
| --- | --- |
| ITS2 Forward Primer Sym_VAR_5.8S2 | TCG TCG GCA GCG TC *AGA TGT GTA TAA GAG ACA G* **NNNN** GAA TTG CAG AAC TCC GTG AAC C |
| ITS2 Reverse Primer SYM_VAR_REV | GTC TCG TGG GCT CGG *AGA TGT GTA TAA GAG ACA G* **NNNN** CGG GTT CWC TTG TYT GAC TTC ATG C |
| 16S Forward Primer 515 F | TCG TCG GCA GCG TC *AGA TGT GTA TAA GAG ACA G* **NNNN** GTG YCA GCM GCC GCG GTA A |
| 16S Reverse Primer 806 R | GTC TCG TGG GCT CGG *AGA TGT GTA TAA GAG ACA G* **NNNN** GGA CTA CNV GGG TWT CTA AT |
| **Barcoding Primer** | Illumina P5 or P7, **Barcode (i5 or i7),** Adapter |
| Hyb_F13_i5 | AAT GAT ACG GCG ACC ACC GAG ATC TAC AC **AGTCAA** T CGT CGG CAG CGT C |
| Hyb_F14_i5 | AAT GAT ACG GCG ACC ACC GAG ATC TAC AC **AGTTCC** T CGT CGG CAG CGT C |
| Hyb_F16_i5 | AAT GAT ACG GCG ACC ACC GAG ATC TAC AC **CCGTCC** T CGT CGG CAG CGT C |
| Hyb_F17_i5 | AAT GAT ACG GCG ACC ACC GAG ATC TAC AC **GTAGAG** T CGT CGG CAG CGT C |
| Hyb_F19_i5 | AAT GAT ACG GCG ACC ACC GAG ATC TAC AC **GTGAAA** T CGT CGG CAG CGT C |
| Hyb_F20_i5 | AAT GAT ACG GCG ACC ACC GAG ATC TAC AC **GTGGCC** T CGT CGG CAG CGT C |
| Hyb_F22_i5 | AAT GAT ACG GCG ACC ACC GAG ATC TAC AC **CGTACG** T CGT CGG CAG CGT C |
| Hyb_F23_i5 | AAT GAT ACG GCG ACC ACC GAG ATC TAC AC **GAGTGG** T CGT CGG CAG CGT C |
| Hyb_F25_i5 | AAT GAT ACG GCG ACC ACC GAG ATC TAC AC **ACTGAT** T CGT CGG CAG CGT C |
| Hyb_F27_i5 | AAT GAT ACG GCG ACC ACC GAG ATC TAC AC **ATTCCT** T CGT CGG CAG CGT C |
| Hyb_F29_i5 | AAT GAT ACG GCG ACC ACC GAG ATC TAC AC **CAACTA** T CGT CGG CAG CGT C |
| Hyb_F31_i5 | AAT GAT ACG GCG ACC ACC GAG ATC TAC AC **CACGAT** T CGT CGG CAG CGT C |
| Hyb_R39_i7 | CAA GCA GAA GAC GGC ATA CGA GAT **GTATAG** GTC TCG TGG GCT CGG |
| Hyb_R40_i7 | CAA GCA GAA GAC GGC ATA CGA GAT **TCTGAG** GTC TCG TGG GCT CGG |
| Hyb_R41_i7 | CAA GCA GAA GAC GGC ATA CGA GAT **GTCGTC** GTC TCG TGG GCT CGG |
| Hyb_R43_i7 | CAA GCA GAA GAC GGC ATA CGA GAT **GCTGTA** GTC TCG TGG GCT CGG |
| Hyb_R44_i7 | CAA GCA GAA GAC GGC ATA CGA GAT **ATTATA** GTC TCG TGG GCT CGG |
| Hyb_R45_i7 | CAA GCA GAA GAC GGC ATA CGA GAT **GAATGA** GTC TCG TGG GCT CGG |
| Hyb_R46_i7 | CAA GCA GAA GAC GGC ATA CGA GAT **TCGGGA** GTC TCG TGG GCT CGG |
| Hyb_R48_i7 | CAA GCA GAA GAC GGC ATA CGA GAT **TGCCGA** GTC TCG TGG GCT CGG |

**Table S3.** Number of samples collected for each species at each timepoint and site. Asterisks denote samples that were cut and transferred into appropriate volumes of RNA later upon returning to CARMABI (field station).

| **Timepoint** | **Site** | ***Siderastrea siderea*** | ***Siderastrea radians*** | **Branching *Porites* sp*.*** |
| --- | --- | --- | --- | --- |
| March 2020 | Santa Martha Bay | 6 | 6 |  |
|  | Santa Martha Reef | 6 |  |  |
|  | Spaanse Water Bay | 6 | 6 |  |
|  | Spaanse Water Reef | 6 |  |  |
| November 2020 | Santa Martha Bay | 6 | 6 |  |
|  | Santa Martha Reef | 6 |  |  |
|  | Spaanse Water Bay | 6 | 8 | 6* |
|  | Spaanse Water Reef | 6 |  | 6* |
| November 2021 | Santa Martha Bay | 10 | 5 |  |
|  | Santa Martha Reef | 10 |  |  |
|  | Spaanse Water Bay | 6 | 5 | 6 |
|  | Spaanse Water Reef | 6 |  | 6 |
| Total | | 80 | 36 | 24 |

**Table S4.** Read counts throughout initial microbiome data processing through the *DADA2* pipeline.

| **ID** | **Full Sample ID** | **Input** | **Filtered** | **Denoised** | **Merged** | **Tabled** | **Non-chimera** |
| --- | --- | --- | --- | --- | --- | --- | --- |
| A1 | 77SSSWBMarch2020 | 45,947 | 45,079 | 42,692 | 33,719 | 33,603 | 32,328 |
| A10 | 82SSSMBMarch2020 | 50,200 | 49,170 | 48,199 | 44,663 | 44,609 | 43,718 |
| A11 | 86SSSMBMarch2020 | 43,263 | 42,285 | 40,887 | 34,938 | 34,897 | 34,779 |
| A12 | 93SSSMBMarch2020 | 75,379 | 74,215 | 73,410 | 70,636 | 70,630 | 69,822 |
| A2 | 78SSSWBMarch2020 | 68,721 | 67,349 | 66,868 | 64,077 | 64,077 | 58,672 |
| A3 | 83SSSWBMarch2020 | 46,975 | 45,569 | 44,601 | 40,925 | 40,624 | 40,423 |
| A4 | 95SSSWBMarch2020 | 79,753 | 77,485 | 75,158 | 66,989 | 66,959 | 66,620 |
| A5 | 13SSDBMarch2020 | 78,073 | 76,249 | 76,037 | 75,051 | 74,862 | 73,099 |
| A6 | 85SSDBMarch2020 | 40,782 | 39,429 | 38,929 | 37,368 | 37,368 | 37,050 |
| A7 | 96SSDBMarch2020 | 54,169 | 52,671 | 51,695 | 48,486 | 48,468 | 46,218 |
| A8 | 97SSDBMarch2020 | 45,402 | 44,157 | 43,650 | 41,556 | 41,556 | 40,087 |
| A9 | 74SSSMBMarch2020 | 111,950 | 110,236 | 109,745 | 107,846 | 107,464 | 106,860 |
| B1 | 61SSSMRMarch2020 | 65,002 | 63,040 | 61,768 | 55,861 | 55,805 | 55,556 |
| B10 | 73SRSMBMarch2020 | 14,525 | 14,119 | 13,060 | 11,161 | 6,631 | 6,631 |
| B11 | 89SRSMBMarch2020 | 20,538 | 19,846 | 18,870 | 16,721 | 16,540 | 16,366 |
| B12 | 90SRSMBMarch2020 | 26,335 | 25,713 | 25,083 | 24,295 | 23,526 | 23,448 |
| B2 | 62SSSMRMarch2020 | 50,268 | 48,897 | 47,500 | 44,246 | 44,233 | 42,954 |
| B3 | 63SSSMRMarch2020 | 39,967 | 38,318 | 36,984 | 32,847 | 32,847 | 32,450 |
| B4 | 64SSSMRMarch2020 | 25,817 | 24,964 | 23,461 | 19,939 | 19,935 | 19,903 |
| B5 | 75SRSWBMarch2020 | 31,566 | 30,366 | 28,573 | 23,938 | 23,919 | 23,737 |
| B6 | 79SRSWBMarch2020 | 51,159 | 48,968 | 45,563 | 34,995 | 34,986 | 34,930 |
| B7 | 80SRSWBMarch2020 | 32,725 | 31,587 | 27,704 | 17,002 | 16,992 | 16,545 |
| B8 | 84SRSWBMarch2020 | 5,471 | 5,260 | 4,798 | 3,788 | 1,851 | 1,851 |
| B9 | 98SRSWBMarch2020 | 20,825 | 20,204 | 17,790 | 13,405 | 10,687 | 10,616 |
| C1 | 92SRSMBMarch2020 | 24,519 | 23,855 | 22,793 | 20,917 | 20,034 | 19,994 |
| C10 | 35SSSMBNovember2020 | 6,277 | 6,120 | 5,330 | 4,105 | 4,081 | 4,046 |
| C11 | 37SSSMBNovember2020 | 5,532 | 5,344 | 4,874 | 4,150 | 3,627 | 3,408 |
| C12 | 70SSSMBNovember2020 | 8,557 | 8,370 | 8,026 | 7,179 | 6,876 | 5,631 |
| C2 | 56SSSWBNovember2020 | 3,964 | 3,823 | 2,738 | 2,033 | 2,019 | 2,019 |
| C3 | 77SSSWBNovember2020 | 5,820 | 5,611 | 4,089 | 2,676 | 2,645 | 2,639 |
| C4 | 83SSSWBNovember2020 | 22,409 | 21,813 | 19,474 | 15,228 | 14,594 | 14,118 |
| C5 | 95SSSWBNovember2020 | 7,078 | 6,894 | 5,328 | 3,608 | 3,520 | 3,520 |
| C6 | 49SSDBNovember2020 | 6,290 | 6,049 | 5,935 | 5,431 | 5,420 | 4,746 |
| C7 | 85SSDBNovember2020 | 6,072 | 5,898 | 5,718 | 5,063 | 5,059 | 4,382 |
| C8 | 96SSDBNovember2020 | 4,862 | 4,683 | 4,376 | 3,885 | 3,852 | 3,685 |
| C9 | 97SSDBNovember2020 | 9,838 | 9,606 | 9,350 | 8,259 | 8,223 | 6,751 |
| D1 | 82SSSMBNovember2020 | 9,582 | 9,332 | 8,340 | 6,557 | 6,033 | 6,010 |
| D10 | 84SRSWBNovember2020 | 13,198 | 12,810 | 11,579 | 10,021 | 9,099 | 9,062 |
| D11 | 98SRSWBNovember2020 | 11,093 | 10,796 | 9,303 | 7,605 | 7,594 | 7,152 |
| D12 | 44SRSMBNovember2020 | 7,653 | 7,451 | 6,913 | 6,059 | 5,992 | 5,159 |
| D2 | 42SSSMRNovember2020 | 27,389 | 26,600 | 25,610 | 24,180 | 21,309 | 20,796 |
| D3 | 43SSSMRNovember2020 | 10,428 | 10,022 | 9,832 | 9,685 | 8,612 | 8,301 |
| D4 | 63SSSMRNovember2020 | 5,113 | 4,972 | 3,991 | 2,713 | 2,668 | 2,652 |
| D5 | 64SSSMRNovember2020 | 25,987 | 25,268 | 22,663 | 18,402 | 18,134 | 16,919 |
| D6 | 91SRSWBNovember2020 | 9,726 | 9,363 | 7,855 | 6,028 | 5,880 | 5,880 |
| D7 | 75SRSWBNovember2020 | 7,763 | 7,538 | 6,498 | 5,581 | 5,251 | 5,242 |
| D8 | 79SRSWBNovember2020 | 4,404 | 4,212 | 3,317 | 2,268 | 2,209 | 2,209 |
| D9 | 80SRSWBNovember2020 | 19,786 | 19,204 | 18,386 | 17,315 | 16,803 | 13,842 |
| F1 | 68PPSMRNovember2020 | 77,727 | 76,455 | 75,213 | 71,326 | 71,320 | 70,981 |
| F10 | 35SSSMBNovember2021 | 48,180 | 47,016 | 46,388 | 44,063 | 38,353 | 36,909 |
| F11 | 37SSSMBNovember2021 | 36,790 | 35,760 | 32,833 | 25,896 | 25,323 | 25,034 |
| F12 | 70SSSMBNovember2021 | 69,476 | 67,718 | 65,760 | 58,860 | 54,855 | 51,331 |
| F2 | 59SSSWBNovember2021 | 60,717 | 58,694 | 58,015 | 53,424 | 53,054 | 42,473 |
| F3 | 77SSSWBNovember2021 | 50,740 | 48,572 | 46,175 | 40,692 | 39,780 | 37,504 |
| F4 | 83SSSWBNovember2021 | 91,521 | 88,931 | 87,832 | 83,110 | 73,166 | 70,550 |
| F5 | 95SSSWBNovember2021 | 57,636 | 56,160 | 54,464 | 49,129 | 48,613 | 48,009 |
| F6 | 69SSDBNovember2021 | 84,741 | 82,422 | 81,929 | 79,747 | 74,642 | 70,598 |
| F7 | 85SSDBNovember2021 | 81,530 | 79,292 | 78,787 | 74,898 | 72,923 | 58,972 |
| F8 | 96SSDBNovember2021 | 44,830 | 43,336 | 42,431 | 39,832 | 38,755 | 33,791 |
| F9 | 97SSDBNovember2021 | 87,246 | 85,148 | 84,555 | 81,273 | 75,801 | 71,251 |
| G1 | 82SSSMBNovember2021 | 33,587 | 32,758 | 31,527 | 29,104 | 26,570 | 26,168 |
| G10 | 44SRSMBNovember2021 | 94,304 | 91,310 | 89,161 | 77,212 | 76,055 | 74,824 |
| G11 | 73SRSMBNovember2021 | 41,618 | 40,577 | 39,615 | 36,830 | 35,516 | 31,088 |
| G12 | 88SRSMBNovember2021 | 42,646 | 41,443 | 40,012 | 37,483 | 37,386 | 36,888 |
| G2 | 42SSSMRNovember2021 | 54,707 | 53,088 | 52,132 | 48,089 | 44,927 | 43,501 |
| G3 | 63SSSMRNovember2021 | 37,078 | 35,951 | 34,325 | 31,688 | 27,205 | 26,329 |
| G4 | 64SSSMRNovember2021 | 46,804 | 45,577 | 44,995 | 43,008 | 37,740 | 35,367 |
| G5 | 72SSSMRNovember2021 | 62,576 | 61,032 | 59,474 | 56,048 | 48,950 | 48,111 |
| G6 | 75SRSWBNovember2021 | 40,743 | 39,132 | 37,315 | 31,447 | 31,107 | 30,537 |
| G7 | 80SRSWBNovember2021 | 26,027 | 25,065 | 23,295 | 19,341 | 19,099 | 18,928 |
| G8 | 84SRSWBNovember2021 | 64,799 | 61,581 | 59,293 | 51,268 | 51,160 | 48,186 |
| G9 | 98SRSWBNovember2021 | 48,646 | 47,047 | 46,481 | 37,757 | 36,362 | 36,042 |
| H1 | 95SRSMBNovember2021 | 40,800 | 39,816 | 37,738 | 33,726 | 32,422 | 32,171 |
| H10 | 47PPSMRNovember2021 | 86,353 | 84,939 | 84,506 | 83,595 | 83,549 | 83,548 |
| H11 | 52PPSMRNovember2021 | 26,413 | 26,033 | 25,985 | 25,790 | 25,630 | 25,630 |
| H12 | blank | 2,257 | 2,224 | 2,194 | 2,194 | 2,194 | 2,194 |
| H2 | 7PPSMBNovember2021 | 36,749 | 35,974 | 34,934 | 31,942 | 31,934 | 31,863 |
| H3 | 11PPSMBNovember2021 | 27,996 | 27,216 | 27,152 | 26,852 | 26,834 | 26,834 |
| H4 | 24PPSMBNovember2021 | 75,812 | 73,868 | 73,442 | 69,507 | 69,489 | 68,585 |
| H5 | 25PPSMBNovember2021 | 62,827 | 62,012 | 61,941 | 61,730 | 61,354 | 61,354 |
| H6 | 50PPSMBNovember2021 | 62,148 | 59,188 | 58,546 | 55,531 | 55,516 | 53,503 |
| H7 | 3PPSMRNovember2021 | 87,464 | 85,248 | 84,952 | 83,620 | 83,544 | 82,946 |
| H8 | 4PPSMRNovember2021 | 28,175 | 27,567 | 27,509 | 27,225 | 27,111 | 27,084 |
| H9 | 28PPSMRNovember2021 | 36,075 | 35,618 | 35,350 | 34,882 | 34,852 | 34,774 |
| I10 | 71SSSWBMarch2020 | 165,807 | 159,947 | 156,363 | 142,539 | 140,078 | 126,807 |
| I11 | 88SSSWBMarch2020 | 134,909 | 130,715 | 125,232 | 104,386 | 103,963 | 100,009 |
| I12 | 1SSDBMarch2020 | 214,296 | 209,345 | 208,884 | 201,446 | 197,328 | 147,499 |
| J1 | 21SSDBMarch2020 | 30,697 | 29,581 | 29,344 | 27,196 | 26,408 | 26,141 |
| J10 | 2PASWBMarch2020 | 116,565 | 112,238 | 110,933 | 103,450 | 96,399 | 94,165 |
| J11 | 3PASWBMarch2020 | 129,307 | 125,723 | 120,836 | 96,701 | 87,783 | 71,024 |
| J12 | 2PADBMarch2020 | 131,826 | 128,513 | 128,276 | 127,167 | 102,581 | 100,795 |
| J2 | 14SSSMBMarch2020 | 136,831 | 132,441 | 131,641 | 124,620 | 115,797 | 102,741 |
| J3 | 100SSSMBMarch2020 | 92,911 | 89,400 | 87,057 | 78,004 | 71,312 | 70,669 |
| J4 | 3SSSMRMarch2020 | 189,458 | 184,198 | 182,988 | 173,760 | 166,096 | 151,780 |
| J5 | 65SSSMRMarch2020 | 114,167 | 111,139 | 105,747 | 80,477 | 79,937 | 72,324 |
| J6 | 9SRSWBMarch2020 | 112,412 | 107,295 | 103,447 | 88,921 | 86,629 | 84,107 |
| J7 | 81SRSMBMarch2020 | 20,380 | 19,706 | 18,038 | 15,865 | 13,325 | 13,325 |
| J8 | 94SRSMBMarch2020 | 91,295 | 88,506 | 87,653 | 82,578 | 77,578 | 74,169 |
| J9 | 1PASWBMarch2020 | 111,889 | 109,298 | 107,481 | 102,584 | 67,220 | 63,423 |
| K1 | 12PADBMarch2020 | 109,551 | 107,032 | 106,661 | 105,788 | 58,265 | 50,228 |
| K10 | 72SSSMRNovember2020 | 137,609 | 134,213 | 129,967 | 110,042 | 104,658 | 100,387 |
| K11 | 58SRSWBNovember2020 | 83,514 | 80,887 | 75,233 | 58,500 | 57,743 | 53,931 |
| K12 | 9SRSWBNovember2020 | 161,975 | 157,290 | 150,412 | 109,206 | 107,202 | 99,284 |
| K2 | 87PADBMarch2020 | 106,919 | 103,948 | 103,292 | 100,893 | 62,324 | 58,690 |
| K3 | XXSSSWBNovember2020 | 44,446 | 42,809 | 38,881 | 31,079 | 30,413 | 27,990 |
| K4 | 71SSSWBNovember2020 | 3,925 | 3,774 | 3,072 | 2,579 | 2,307 | 2,307 |
| K5 | 10SSDBNovember2020 | 116,276 | 112,308 | 111,160 | 102,481 | 93,889 | 80,121 |
| K6 | 41SSDBNovember2020 | 86,581 | 82,511 | 81,885 | 74,784 | 74,359 | 53,480 |
| K7 | 8SSSMBNovember2020 | 118,297 | 114,931 | 112,844 | 102,428 | 97,200 | 93,056 |
| K8 | 40SSSMBNovember2020 | 30,715 | 28,805 | 27,772 | 24,705 | 24,272 | 23,626 |
| K9 | 48SSSMRNovember2020 | 703 | 654 | 449 | 403 | 352 | 352 |
| L1 | 91SRSWBNovember2020 | 113,446 | 109,523 | 104,357 | 85,035 | 82,191 | 79,475 |
| L10 | 8SSSMBNovember2021 | 7,368 | 7,088 | 6,534 | 5,623 | 5,451 | 4,369 |
| L11 | 18SSSMBNovember2021 | 192,509 | 186,849 | 183,288 | 163,798 | 163,645 | 119,582 |
| L12 | 33SSSMBNovember2021 | 69,502 | 67,975 | 64,846 | 55,206 | 54,651 | 50,256 |
| L2 | 36SRSMBNovember2020 | 32,987 | 31,582 | 29,563 | 23,899 | 23,213 | 20,609 |
| L3 | 45SRSMBNovember2020 | 5,978 | 5,700 | 4,887 | 4,129 | 4,095 | 3,620 |
| L4 | 15PPSMBNovember2020 | 192,539 | 186,452 | 184,611 | 174,455 | 157,123 | 146,998 |
| L5 | 7PPSMRNovember2020 | 191,578 | 187,767 | 183,978 | 170,603 | 89,535 | 77,234 |
| L6 | 71SSSWBNovember2021 | 9,919 | 9,427 | 9,153 | 7,821 | 7,721 | 4,729 |
| L7 | 94SSSWBNovember2021 | 67,218 | 65,000 | 63,031 | 55,916 | 55,480 | 47,040 |
| L8 | 38SSDBNovember2021 | 5,324 | 5,096 | 4,672 | 4,218 | 3,901 | 3,821 |
| L9 | 53SSDBNovember2021 | 19,935 | 19,409 | 19,037 | 18,107 | 16,866 | 15,403 |
| M1 | 39SSSMBNovember2021 | 9,776 | 9,344 | 8,384 | 6,640 | 6,280 | 5,962 |
| M10 | 86SRSMBNovember2021 | 51,314 | 49,441 | 47,463 | 40,256 | 40,121 | 31,897 |
| M11 | 26PPSMBNovember2021 | 167,792 | 163,734 | 163,578 | 162,289 | 79,474 | 79,461 |
| M12 | 97PPSMBNovember2021 | 191,064 | 186,637 | 186,367 | 185,470 | 61,272 | 61,147 |
| M2 | 44SSSMBNovember2021 | 3,576 | 3,426 | 3,181 | 2,706 | 2,471 | 2,112 |
| M3 | 99SSSMBNovember2021 | 685 | 627 | 446 | 389 | 295 | 295 |
| M4 | 15SSSMRNovember2021 | 11,129 | 10,789 | 10,461 | 9,959 | 8,480 | 7,682 |
| M5 | 16SSSMRNovember2021 | 1,960 | 1,850 | 1,509 | 1,349 | 1,109 | 1,109 |
| M6 | 17SSSMRNovember2021 | 892 | 818 | 617 | 455 | 387 | 387 |
| M7 | 18SSSMRNovember2021 | 115,675 | 111,439 | 109,293 | 101,622 | 100,456 | 91,664 |
| M8 | 19SSSMRNovember2021 | 45,959 | 44,082 | 43,179 | 39,698 | 39,153 | 34,913 |
| M9 | 56SRSWBNovember2021 | 956 | 887 | 741 | 560 | 491 | 444 |
| N1 | 7PPSMBNovember2021 | 112,927 | 110,609 | 107,744 | 99,710 | 48,404 | 46,557 |
| N10 | 52PPSMRNovember2021 | 52,841 | 51,782 | 51,481 | 51,082 | 16,378 | 16,177 |
| N12 | blank | 373 | 307 | 235 | 219 | 166 | 166 |
| N2 | 11PPSMBNovember2021 | 71,601 | 69,769 | 69,401 | 67,703 | 20,100 | 17,918 |
| N3 | 24PPSMBNovember2021 | 93,666 | 90,366 | 89,313 | 83,877 | 47,437 | 40,863 |
| N4 | 25PPSMBNovember2021 | 128,219 | 125,795 | 125,475 | 125,030 | 43,474 | 43,474 |
| N5 | 50PPSMBNovember2021 | 102,072 | 99,689 | 98,200 | 93,075 | 47,928 | 39,660 |
| N6 | 3PPSMRNovember2021 | 72,170 | 68,593 | 67,947 | 65,799 | 33,414 | 30,210 |
| N7 | 4PPSMRNovember2021 | 112,321 | 110,109 | 109,738 | 108,628 | 32,685 | 30,488 |
| N8 | 28PPSMRNovember2021 | 31,649 | 30,844 | 30,638 | 30,459 | 13,417 | 12,531 |
| N9 | 47PPSMRNovember2021 | 138,930 | 136,402 | 135,614 | 133,313 | 56,957 | 53,452 |

**Table S5. A.** Symbiodiniaceae community summary statistics examining the proportion of samples dominated by (> 70%) a majority ITS2 type. The proportion of samples dominated by majority ITS2 types is shown across coral species, and then within coral species comparisons of habitat or location, timepoint, and site (habitat:location interaction). **B.** Multinomial model results of these comparisons of dominant majority ITS2 types and post-hoc emmeans comparisons. n.s. = not significant, SM = Santa Martha, SW = Spaanse Water.

| 1. **Symbiodiniaceae community summary statistics** | | | | | | | | | |
| --- | --- | --- | --- | --- | --- | --- | --- | --- | --- |
| **Coral Species** |  |  |  |  |  |  |  |  |  |
| **Variable** | **Levels** | **No. samples** | **Prop. dominated by Majority ITS2 Type** | | | | | | |
|  |  |  | **A4** | **C1** | **C3** | **C42** | **C46** | **C47a** | **D1** |
| **Coral Species** | *S. siderea* | 78 | 0 | 0.09 | 0.24 | 0 | 0 | 0 | 0.55 |
|  | *S. radians* | 36 | 0 | 0.05 | 0 | 0 | 0.89 | 0 | 0.03 |
|  | *Porites* sp | 23 | 0.52 | 0 | 0 | 0.17 | 0 | 0.22 | 0 |
| ***Siderastrea siderea*** | | | | | | | | | |
| **Variable** | **Levels** | **No. samples** | **Prop. dominated by Majority ITS2 Type** | | | | | | |
|  |  |  | **A4** | **C1** | **C3** | **C42** | **C46** | **C47a** | **D1** |
| **Timepoint** | March 2020 | 24 |  | 0.13 | 0.25 |  |  |  | 0.5 |
|  | November 2020 | 24 |  | 0.04 | 0.25 |  |  |  | 0.54 |
|  | November 2021 | 30 |  | 0.1 | 0.23 |  |  |  | 0.6 |
| **Habitat** | Bay | 40 |  | 0.08 | 0 |  |  |  | 0.83 |
|  | Reef | 38 |  | 0.11 | 0.5 |  |  |  | 0.26 |
| **Site (Habitat: Location)** | SM_bay | 22 |  | 0.09 | 0 |  |  |  | 0.77 |
|  | SM_reef | 21 |  | 0.19 | 0.38 |  |  |  | 0.29 |
|  | SW_bay | 18 |  | 0.06 | 0 |  |  |  | 0.89 |
|  | SW_reef | 17 |  | 0 | 0.65 |  |  |  | 0.24 |
| ***Siderastrea radians*** | | | | | | | | | |
| **Variable** | **Levels** | **No. samples** | **Prop. dominated by Majority ITS2 Type** | | | | | | |
|  |  |  | **A4** | **C1** | **C3** | **C42** | **C46** | **C47a** | **D1** |
| **Timepoint** | March 2020 | 12 |  | 0 |  |  | 1 |  | 0 |
|  | November 2020 | 14 |  | 0.14 |  |  | 0.71 |  | 0.07 |
|  | November 2021 | 10 |  | 0 |  |  | 1 |  | 0 |
| **Location** | SM_bay | 17 |  | 0.12 |  |  | 0.88 |  | 0 |
|  | SW_bay | 19 |  | 0 |  |  | 0.9 |  | 0.05 |
| **Branching *Porites* sp.** | | | | | | | | | |
| **Variable** | **Levels** | **No. samples** | **Prop. dominated by Majority ITS2 Type** | | | | | | |
|  |  |  | **A4** | **C1** | **C3** | **C42** | **C46** | **C47a** | **D1** |
| **Timepoint** | November 2020 | 11 | 0.55 |  |  | 0.18 |  | 0.09 |  |
|  | November 2021 | 12 | 0.5 |  |  | 0.17 |  | 0.33 |  |
| **Habitat** | SM_bay | 12 | 1 |  |  | 0 |  | 0 |  |
|  | SM_reef | 11 | 0 |  |  | 0.36 |  | 0.46 |  |

| **B. Symbiodiniaceae community multinomial statistics** | | | | | | | | |
| --- | --- | --- | --- | --- | --- | --- | --- | --- |
| **Coral Species** | | | | | | | | |
| **Variable** | **Model** |  | **LR chisq** |  | **df** |  | **p-value** | **significance** |
| **Coral Species** | Dominant ITS2 Type ~ Species |  | 226.95 |  | 12 |  | <0.001 | *** |
|  | Dominant **ITS2 Type** | **Contrast** | **Estimate** | **SE** | **df** | **t-ratio** | **p-value** | **significance** |
|  | **A4** | *Porites* sp. *- S. radians* | 0.57 | 0.11 | 18 | 5.29 | 0.0001 | *** |
|  |  | *Porites sp. - S. siderea* | 0.57 | 0.11 | 18 | 5.29 | 0.0001 | *** |
|  |  | *S. radians - S. siderea* | 0.00 | 0.00 | 18 | 2.34 | 0.0750 | n.s. |
|  | **C1** | *Porites* sp. *- S. radians* | -0.06 | 0.04 | 18 | -1.46 | 0.3345 | n.s. |
|  |  | *Porites sp. - S. siderea* | -0.10 | 0.04 | 18 | -2.79 | 0.0309 | * |
|  |  | *S. radians - S. siderea* | -0.04 | 0.05 | 18 | -0.83 | 0.6907 | n.s. |
|  | **C3** | *Porites* sp. *- S. radians* | 0.00 | 0.00 | 18 | 4.56 | 0.0007 | *** |
|  |  | *Porites sp. - S. siderea* | -0.28 | 0.05 | 18 | -5.12 | 0.0002 | *** |
|  |  | *S. radians - S. siderea* | -0.28 | 0.05 | 18 | -5.12 | 0.0002 | *** |
|  | **C42** | *Porites* sp. *- S. radians* | 0.19 | 0.09 | 18 | 2.22 | 0.0942 | n.s. |
|  |  | *Porites sp. - S. siderea* | 0.19 | 0.09 | 18 | 2.22 | 0.0942 | n.s. |
|  |  | *S. radians - S. siderea* | 0.00 | 0.00 | 18 | -1.69 | 0.2380 | n.s. |
|  | **C46** | *Porites* sp. *- S. radians* | -0.91 | 0.05 | 18 | -19.32 | <.0001 | *** |
|  |  | *Porites sp. - S. siderea* | 0.00 | 0.00 | 18 | 3.44 | 0.0079 | n.s. |
|  |  | *S. radians - S. siderea* | 0.91 | 0.05 | 18 | 19.32 | <.0001 | *** |
|  | **C47a** | *Porites* sp. *- S. radians* | 0.24 | 0.09 | 18 | 2.56 | 0.0491 | * |
|  |  | *Porites sp. - S. siderea* | 0.24 | 0.09 | 18 | 2.56 | 0.0491 | * |
|  |  | *S. radians - S. siderea* | 0.00 | 0.00 | 18 | -1.83 | 0.1886 | n.s. |
|  | **D1** | *Porites* sp. *- S. radians* | -0.03 | 0.03 | 18 | -1.02 | 0.5775 | n.s. |
|  |  | *Porites sp. - S. siderea* | -0.62 | 0.06 | 18 | -10.68 | <.0001 | *** |
|  |  | *S. radians - S. siderea* | -0.59 | 0.06 | 18 | -9.18 | <.0001 | *** |
| ***Siderastrea siderea*** | | | | | | | | |
| **Variable** | **Model** | **contrast** | **LR chisq** |  | **df** |  | **p-value** | **significance** |
| **Timepoint** | Dominant ITS2 Type ~ Timepoint | timepoint | 1.2175 |  | 4 |  | 0.8752 | n.s. |
| **Habitat** | Dominant ITS2 Type ~ Habitat | habitat | 39.32 |  | 2 |  | <0.001 | *** |
|  | **ITS2 Type** | **contrast** | **estimate** | **SE** | **df** | **t.ratio** | **p.value** | **significance** |
|  | **C1** | bay - reef | -0.04 | 0.07 | 4 | -0.52 | 0.6316 | n.s. |
|  | **C3** | bay - reef | -0.58 | 0.09 | 4 | -6.69 | 0.0026 | ** |
|  | **D1** | bay - reef | 0.61 | 0.09 | 4 | 6.65 | 0.0027 | ** |
| **Variable** | **Model** | **contrast** | **LR chisq** |  | **df** |  | **p-value** | **significance** |
| **Site** | Dominant ITS2 Type ~ Habitat* Location | habitat: location | 1.4324 |  | 2 |  | 0.48861 | n.s. |
| ***Siderastrea radians*** | | | | | | | | |
| **Variable** | **Model** | **contrast** | **LR chisq** |  | **df** |  | **p-value** | **significance** |
| **Timepoint** | Dominant ITS2 Type ~ Timepoint | timepoint | 6.4302 |  | 4 |  | 0.1692 | n.s. |
| **Location** | Dominant ITS2 Type ~ Location | location | 4.25 |  | 2 |  | 0.12 | n.s. |
| **Branching *Porites* sp.** | | | | | | | | |
| **Variable** | **Model** | **contrast** | **LR chisq** |  | **df** |  | **p-value** | **significance** |
| **Timepoint** | Dominant ITS2 Type ~ Timepoint | timepoint | 1.4974 |  | 2 |  | 0.473 | n.s. |
| **Habitat** | Dominant ITS2 Type ~ Habitat | habitat | 28.68 |  | 2 |  | <0.001 | *** |
|  | **ITS2 Type** | **contrast** | **estimate** | **SE** | **df** | **t.ratio** | **p.value** | **significance** |
|  | **A4** | bay - reef | 1.0000 | 0.0029 | 4 | 339.95 | <.0001 | *** |
|  | **C42** | bay - reef | -0.4440 | 0.1660 | 4 | -2.68 | 0.0551 | n.s. |
|  | **C47a** | bay - reef | -0.5560 | 0.1660 | 4 | -3.35 | 0.0285 | * |

**Table S6.** Alpha diversity metrics of bacterial communities compared across timepoint, habitat, location, and site (habitat:location) comparisons for all coral species, with a random effect of coral genotype using generalized linear models. The lowest AICc models were selected for use and are shown here. n.s = not significant. Mar. = March, Nov. = November, SM = Santa Martha, SW = Spaanse Water, B = Bay, R = Reef.

| **Coral Species** | | | | | | | | |
| --- | --- | --- | --- | --- | --- | --- | --- | --- |
| **Index** | **Model** |  |  |  |  |  |  |  |
| All | Null |  |  |  |  |  |  |  |
| ***Siderastrea siderea*** | | | | | | | | |
| **Index** | **Model** | **Variable** | **Chisq** |  | **Df** |  | **p** | **sig.** |
| Shannon | Shannon ~ Timepoint + Habitat * Location + (1\|Site:Genotype) | (Intercept) | 284.521 |  | 1 |  | <2.20E-16 | *** |
|  |  | Timepoint | 22.9844 |  | 2 |  | 1E-05 | *** |
|  |  | Habitat | 0.5581 |  | 1 |  | 0.455 | n.s. |
|  |  | Location | 0.0044 |  | 1 |  | 0.947 | n.s. |
|  |  | Site | 6.1848 |  | 1 |  | 0.013 | * |
|  | **Variable** | **Contrast** | **Estimate** | **SE** | **Df** | **T-ratio** | **p-value** | **sig.** |
|  | Timepoint | Mar. '20 - Nov. '20 | 1.271 | 0.27 | 66 | 4.669 | <.0001 | *** |
|  |  | Mar. '20 - Nov. '21 | 0.876 | 0.25 | 66 | 3.51 | 0.002 | ** |
|  |  | Nov. '20 - Nov. '21 | -0.395 | 0.25 | 66 | -1.604 | 0.251 | n.s. |
|  | Site | SMB - SMR | -0.2779 | 0.37 | 66 | -0.747 | 0.878 | n.s. |
|  |  | SMB - SWB | -0.0256 | 0.39 | 66 | -0.066 | 1 | n.s. |
|  |  | SMB - SWR | 1.0777 | 0.37 | 66 | 2.883 | 0.027 | * |
|  |  | SMR - SWB | 0.2523 | 0.4 | 66 | 0.626 | 0.924 | n.s. |
|  |  | SMR - SWR | 1.3556 | 0.4 | 66 | 3.424 | 0.006 | ** |
|  |  | SWB - SWR | 1.1033 | 0.41 | 66 | 2.704 | 0.042 | * |
| **Index** | **Model** | **Variable** | **Chisq** |  | **Df** |  | **p-value** | **sig.** |
| Inverse Simpson | log(Inverse Simpson) ~ Timepoint + Habitat * Location + (1\|Site:Genotype) | (Intercept) | 179.261 |  | 1 |  | <2.00E-16 | *** |
|  |  | Timepoint | 9.3662 |  | 2 |  | 0.009 | ** |
|  |  | Habitat | 0.677 |  | 1 |  | 0.411 | n.s. |
|  |  | Location | 0.2185 |  | 1 |  | 0.64 | n.s. |
|  |  | Site | 5.4691 |  | 1 |  | 0.019 | * |
|  | **Variable** | **Contrast** | **Estimate** | **SE** | **Df** | **T-ratio** | **p-value** | **sig.** |
|  | Timepoint | Mar. '20 - Nov. '20 | 0.822 | 0.28 | 66 | 2.925 | 0.013 | * |
|  |  | Mar. '20 - Nov. '21 | 0.587 | 0.25 | 66 | 2.333 | 0.058 | n.s. |
|  |  | Nov. '20 - Nov. '21 | -0.235 | 0.26 | 66 | -0.912 | 0.635 | n.s. |
|  | Site | SMB - SMR | -0.268 | 0.33 | 66 | -0.823 | 0.844 | n.s. |
|  |  | SMB - SWB | -0.155 | 0.33 | 66 | -0.467 | 0.966 | n.s. |
|  |  | SMB - SWR | 0.732 | 0.33 | 66 | 2.239 | 0.124 | n.s. |
|  |  | SMR - SWB | 0.113 | 0.34 | 66 | 0.327 | 0.988 | n.s. |
|  |  | SMR - SWR | 0.999 | 0.36 | 66 | 2.816 | 0.032 | * |
|  |  | SWB - SWR | 0.887 | 0.36 | 66 | 2.485 | 0.072 | n.s. |
| **Index** | **Model** | **Variable** | **Chisq** |  | **Df** |  | **p-value** | **sig.** |
| Richness | log(Richness) ~ Timepoint + Habitat * Location + (1\|Site:Genotype) | (Intercept) | 719.839 |  | 1 |  | <2.20E-16 | *** |
|  |  | Timepoint | 29.8453 |  | 2 |  | 3E-07 | *** |
|  |  | Habitat | 0.5572 |  | 1 |  | 0.455 | n.s. |
|  |  | Location | 0.034 |  | 1 |  | 0.854 | n.s. |
|  |  | Site | 6.6554 |  | 1 |  | 0.01 | ** |
|  | **Variable** | **Contrast** | **Estimate** | **SE** | **Df** | **T-ratio** | **p-value** | **sig.** |
|  | Timepoint | Mar. '20 - Nov. '20 | 1.389 | 0.26 | 66 | 5.403 | <.0001 | *** |
|  |  | Mar. '20 - Nov. '21 | 0.833 | 0.24 | 66 | 3.434 | 0.003 | ** |
|  |  | Nov. '20 - Nov. '21 | -0.555 | 0.25 | 66 | -2.235 | 0.073 | n.s. |
|  | Site | SMB - SMR | -0.2088 | 0.28 | 66 | -0.746 | 0.878 | n.s. |
|  |  | SMB - SWB | 0.0525 | 0.28 | 66 | 0.184 | 0.998 | n.s. |
|  |  | SMB - SWR | 0.8916 | 0.28 | 66 | 3.181 | 0.012 | * |
|  |  | SMR - SWB | 0.2613 | 0.29 | 66 | 0.887 | 0.811 | n.s. |
|  |  | SMR - SWR | 1.1005 | 0.29 | 66 | 3.789 | 0.002 | ** |
|  |  | SWB - SWR | 0.8392 | 0.29 | 66 | 2.85 | 0.029 | * |
| **Index** | **Model** |  |  |  |  |  |  |  |
| Evenness | Null |  |  |  |  |  |  |  |
| **Index** | **Model** | **Variable** | **Chisq** |  | **Df** |  | **p-value** | **sig.** |
| Phylogenetic Diversity (Faith's D) | log(Faith's D) ~ Timepoint + Habitat * Location + (1\|Site:Genotype) | (Intercept) | 914.37 |  | 1 |  | <2.20E-16 | *** |
|  |  | Timepoint | 16.3963 |  | 2 |  | 3E-04 | *** |
|  |  | Habitat | 0.0949 |  | 1 |  | 0.758 | n.s. |
|  |  | Location | 0.5847 |  | 1 |  | 0.444 | n.s. |
|  |  | Site | 3.5478 |  | 1 |  | 0.06 | n.s. |
|  | **Variable** | **Contrast** | **Estimate** | **SE** | **Df** | **T-ratio** | **p-value** | **sig.** |
|  | Timepoint | Mar. '20 - Nov. '20 | 0.495 | 0.12 | 66 | 4.016 | 4E-04 | *** |
|  |  | Mar. '20 - Nov. '21 | 0.185 | 0.12 | 66 | 1.586 | 0.259 | n.s. |
|  |  | Nov. '20 - Nov. '21 | -0.31 | 0.12 | 66 | -2.605 | 0.03 | * |
| ***Siderastrea radians*** | | | | | | | | |
| **Index** | **Model** |  |  |  |  |  |  |  |
| Shannon | Null |  |  |  |  |  |  |  |
| **Index** | **Model** |  |  |  |  |  |  |  |
| Inverse Simpson | Null |  |  |  |  |  |  |  |
| **Index** | **Model** |  |  |  |  |  |  |  |
| Richness | Null |  |  |  |  |  |  |  |
| **Index** | **Model** |  |  |  |  |  |  |  |
| Evenness | Null |  |  |  |  |  |  |  |
| **Index** | **Model** | **Variable** | **Chisq** |  | **Df** |  | **p-value** | **sig.** |
| Phylogenetic Diversity (Faith's D) | log(Faith's D) ~ Location + Timepoint + (1\|Site:Genotype) | (Intercept) | 983.147 |  | 1 |  | <2.00E-16 | *** |
|  |  | Location | 3.8748 |  | 1 |  | 0.049 | * |
|  |  | Timepoint | 8.5816 |  | 2 |  | 0.014 | * |
|  | **Variable** | **Contrast** | **Estimate** | **SE** | **Df** | **T-ratio** | **p-value** | **sig.** |
|  | Timepoint | Mar. '20 - Nov. '20 | 0.367 | 0.14 | 26 | 2.578 | 0.041 | * |
|  |  | Mar. '20 - Nov. '21 | -0.0401 | 0.14 | 26 | -0.291 | 0.955 | n.s. |
|  |  | Nov. '20 - Nov. '21 | -0.4071 | 0.16 | 26 | -2.619 | 0.037 | * |
|  | Location | SM - SW | -0.239 | 0.12 | 26 | -1.968 | 0.06 | n.s. |
| **Branching *Porites* sp.** | | | | | | | | |
| **Index** | **Model** |  |  |  |  |  |  |  |
| Shannon | Null |  |  |  |  |  |  |  |
| **Index** | **Model** |  |  |  |  |  |  |  |
| Inverse Simpson | Null |  |  |  |  |  |  |  |
| **Index** | **Model** | **Variable** | **Chisq** |  | **Df** |  | **p-value** | **sig.** |
| Richness | log(Richness) ~ Habitat + Timepoint + (1\|Site:Genotype) | (Intercept) | 205.294 |  | 1 |  | <2.00E-16 | *** |
|  |  | Habitat | 0.3712 |  | 1 |  | 0.542 | n.s. |
|  |  | Timepoint | 11.8117 |  | 1 |  | 6E-04 | *** |
|  | **Variable** | **Contrast** | **Estimate** | **SE** | **Df** | **T-ratio** | **p-value** | **sig.** |
|  | Timepoint | Nov. '20 - Nov. '21 | 1.64 | 0.48 | 11 | 3.437 | 0.006 | ** |
|  | Habitat | Reef - Bay | 0.233 | 0.38 | 11 | 0.609 | 0.555 | n.s. |
| **Index** | **Model** |  |  |  |  |  |  |  |
| Evenness | Null |  |  |  |  |  |  |  |
| **Index** | **Model** | **Variable** | **Chisq** |  | **Df** |  | **p-value** | **sig.** |
| Phylogenetic Diversity (Faith's D) | log(Faith's D) ~ Timepoint + (1\|Site:Genotype) | (Intercept) | 407.298 |  | 1 |  | <2.00E-16 | *** |
|  |  | Timepoint | 12.505 |  | 1 |  | 4E-04 | *** |
|  | **Variable** | **Contrast** | **Estimate** | **SE** | **Df** | **T-ratio** | **p-value** | **sig.** |
|  | Timepoint | Nov. '20 - Nov. '21 | 0.705 | 0.2 | 12 | 3.536 | 0.004 | ** |

**Table S7.** Results from Pairwise Permutational Multivariate Analysis of Variance (PERMANOVA) of Bray-Curtis distances to assess differences in bacterial beta diversity between coral species and within coral species differences between habitats, timepoints, Sites, habitat:timepoints (or location:timepoint for S. radians), and Site:timepoints. Mar. = March, Nov. = November, SM = Santa Martha, SW = Spaanse Water, B = Bay, R = Reef. n.s = not significant.

| **Coral species** | | | | | | | |
| --- | --- | --- | --- | --- | --- | --- | --- |
| **Multivariate Homogeneity of Groups Dispersions (betadisper)** | | | | | | | |
| **Variable** |  | **Df** | **Sum Sq** | **Mean Sq** | **F-value** | **p-value** | **sig.** |
| Coral species |  | 2 | 0.00649 | 0.0032468 | 0.9284 | 0.398 | n.s. |
| **Permutational Multivariate Analysis of Variance (adonis)** | | | | | | | |
| **Variable** |  | **Df** | **SumOfSqs** | **R2** | **F-value** | **p-value** | **sig.** |
| Coral species |  | 2 | 1.875 | 0.03681 | 2.2931 | 0.001 | *** |
| **Pairwise adonis** | | | | | | | |
| **Variable** | **Comparison** | **Df** | **SumOfSqs** | **R2** | **F-value** | **p-value** | **sig.** |
| Coral species | *S. siderea* vs *S. radians* | 1 | 1.146 | 0.02629 | 2.8354 | 0.001 | *** |
|  | *S. siderea* vs *Porites* sp. | 1 | 0.689 | 0.01861 | 1.6687 | 0.006 | ** |
|  | *S. radians* vs *Porites* sp. | 1 | 0.9375 | 0.04626 | 2.2797 | 0.001 | *** |
| ***Siderastrea siderea*** | | | | | | | |
| **Multivariate Homogeneity of Groups Dispersions (betadisper)** | | | | | | | |
| **Variable** |  | **Df** | **Sum Sq** | **Mean Sq** | **F-value** | **p-value** | **sig.** |
| Habitat |  | 1 | 0.00348 | 0.0034832 | 0.376 | 0.5417 | n.s. |
| Timepoint |  | 2 | 0.16458 | 0.082291 | 7.7667 | 0.000891 | *** |
| Site |  | 3 | 0.10886 | 0.036286 | 2.5658 | 0.06142 | n.s. |
| Habitat: Timepoint |  | 5 | 0.23305 | 0.046609 | 3.5453 | 0.006591 | ** |
| Site: Timepoint |  | 11 | 0.64209 | 0.058372 | 7.9688 | 1.96E-08 | *** |
| **Permutational Multivariate Analysis of Variance (adonis)** | | | | | | | |
| **Variable** |  | **Df** | **SumOfSqs** | **R2** | **F-value** | **p-value** | **sig.** |
| Habitat |  | 1 | 0.79 | 0.02867 | 2.4674 | 0.003 | ** |
| Timepoint |  | 2 | 2.0931 | 0.07597 | 3.2689 | 0.001 | *** |
| Site |  | 2 | 1.9265 | 0.06992 | 3.0086 | 0.001 | *** |
| Habitat: Timepoint |  | 2 | 0.9136 | 0.03316 | 1.4267 | 0.016 | * |
| Site: Timepoint |  | 4 | 1.9783 | 0.0718 | 1.5448 | 0.001 | *** |
| **Pairwise adonis** | | | | | | | |
| **Variable** | **Comparison** | **Df** | **SumOfSqs** | **R2** | **F-value** | **p-value** | **sig.** |
| Habitat | R vs B | 1 | 0.79 | 0.02867 | 2.1254 | 0.003 | ** |
| Timepoint | Mar. '20 vs Nov. '20 | 1 | 1.3191 | 0.07341 | 3.486 | 0.001 | *** |
|  | Mar. '20 vs Nov. '21 | 1 | 1.2579 | 0.06267 | 3.3428 | 0.001 | *** |
|  | Nov. '20 vs Nov. '21 | 1 | 0.5619 | 0.03505 | 1.7433 | 0.016 | * |
| Site | SWB vs SMB | 1 | 0.6001 | 0.04477 | 1.6871 | 0.011 | * |
|  | SWB vs SWR | 1 | 0.7814 | 0.06558 | 2.3159 | 0.002 | ** |
|  | SWB vs SMR | 1 | 0.8358 | 0.06131 | 2.1552 | 0.003 | ** |
|  | SMB vs SWR | 1 | 1.0026 | 0.07694 | 3.0841 | 0.002 | ** |
|  | SMB vs SMR | 1 | 0.9001 | 0.06169 | 2.4325 | 0.001 | *** |
|  | SWR vs SMR | 1 | 1.3355 | 0.09998 | 3.777 | 0.001 | *** |
| Habitat: Timepoint | B Mar. '20 vs R Mar. '20 | 1 | 0.5663 | 0.05665 | 1.3212 | 0.01 | ** |
|  | B Mar. '20 vs B Nov. '20 | 1 | 0.7423 | 0.08606 | 1.9774 | 0.001 | *** |
|  | B Mar. '20 vs R Nov. '20 | 1 | 1.1275 | 0.1325 | 3.2075 | 0.001 | *** |
|  | B Mar. '20 vs B Nov. '21 | 1 | 0.9606 | 0.10013 | 2.7817 | 0.001 | *** |
|  | B Mar. '20 vs R Nov. '21 | 1 | 0.8999 | 0.09449 | 2.4 | 0.001 | *** |
|  | R Mar. '20 vs B Nov. '20 | 1 | 0.8496 | 0.09396 | 2.1777 | 0.001 | *** |
|  | R Mar. '20 vs R Nov. '20 | 1 | 0.9907 | 0.11412 | 2.7051 | 0.002 | ** |
|  | R Mar. '20 vs B Nov. '21 | 1 | 1.0921 | 0.10884 | 3.0533 | 0.001 | *** |
|  | R Mar. '20 vs R Nov. '21 | 1 | 0.7888 | 0.08113 | 2.0309 | 0.003 | ** |
|  | B Nov. '20 vs R Nov. '20 | 1 | 0.5082 | 0.07639 | 1.6541 | 0.023 | * |
|  | B Nov. '20 vs B Nov. '21 | 1 | 0.4624 | 0.05884 | 1.5005 | 0.045 | * |
|  | B Nov. '20 vs R Nov. '21 | 1 | 0.8221 | 0.10014 | 2.4483 | 0.001 | *** |
|  | R Nov. '20 vs B Nov. '21 | 1 | 0.4005 | 0.0549 | 1.3941 | 0.109 | n.s. |
|  | R Nov. '20 vs R Nov. '21 | 1 | 0.6322 | 0.0841 | 2.02 | 0.031 | * |
|  | B Nov. '21 vs R Nov. '21 | 1 | 0.6823 | 0.07737 | 2.1803 | 0.008 | ** |
| Site: Timepoint | Mar. '20 SWB vs Mar. '20 SMB | 1 | 0.6112 | 0.13403 | 1.5477 | 0.003 | ** |
|  | Mar. '20 SWB vs Mar. '20 SWR | 1 | 0.5358 | 0.11016 | 1.238 | 0.04 | * |
|  | Mar. '20 SWB vs Mar. '20 SMR | 1 | 0.5031 | 0.10586 | 1.1839 | 0.016 | * |
|  | Mar. '20 SWB vs Nov. '20 SMB | 1 | 0.6163 | 0.1415 | 1.6483 | 0.009 | ** |
|  | Mar. '20 SWB vs Nov. '20 SWB | 1 | 0.5791 | 0.15201 | 1.6134 | 0.024 | * |
|  | Mar. '20 SWB vs Nov. '20 SWR | 1 | 1.1576 | 0.31259 | 4.5473 | 0.004 | ** |
|  | Mar. '20 SWB vs Nov. '20 SMR | 1 | 0.4616 | 0.11101 | 1.1239 | 0.193 | n.s. |
|  | Mar. '20 SWB vs Nov. '21 SMB | 1 | 0.7291 | 0.14589 | 2.2204 | 0.007 | ** |
|  | Mar. '20 SWB vs Nov. '21 SWB | 1 | 0.7514 | 0.17764 | 2.1601 | 0.003 | ** |
|  | Mar. '20 SWB vs Nov. '21 SWR | 1 | 0.9884 | 0.23971 | 3.1529 | 0.003 | ** |
|  | Mar. '20 SWB vs Nov. '21 SMR | 1 | 0.6417 | 0.13245 | 1.6794 | 0.002 | ** |
|  | Mar. '20 SMB vs Mar. '20 SWR | 1 | 0.6581 | 0.13885 | 1.6124 | 0.004 | ** |
|  | Mar. '20 SMB vs Mar. '20 SMR | 1 | 0.5335 | 0.11759 | 1.3326 | 0.003 | ** |
|  | Mar. '20 SMB vs Nov. '20 SMB | 1 | 0.6227 | 0.15129 | 1.7826 | 0.003 | ** |
|  | Mar. '20 SMB vs Nov. '20 SWB | 1 | 0.8309 | 0.21779 | 2.5059 | 0.003 | ** |
|  | Mar. '20 SMB vs Nov. '20 SWR | 1 | 1.39 | 0.37674 | 6.0445 | 0.005 | ** |
|  | Mar. '20 SMB vs Nov. '20 SMR | 1 | 0.5721 | 0.14222 | 1.4921 | 0.006 | ** |
|  | Mar. '20 SMB vs Nov. '21 SMB | 1 | 0.7622 | 0.1593 | 2.4634 | 0.003 | ** |
|  | Mar. '20 SMB vs Nov. '21 SWB | 1 | 0.8298 | 0.20426 | 2.5669 | 0.005 | ** |
|  | Mar. '20 SMB vs Nov. '21 SWR | 1 | 1.0742 | 0.27105 | 3.7184 | 0.004 | ** |
|  | Mar. '20 SMB vs Nov. '21 SMR | 1 | 0.6454 | 0.14023 | 1.7942 | 0.001 | *** |
|  | Mar. '20 SWR vs Mar. '20 SMR | 1 | 0.4868 | 0.09998 | 1.1108 | 0.128 | n.s. |
|  | Mar. '20 SWR vs Nov. '20 SMB | 1 | 0.559 | 0.12615 | 1.4436 | 0.026 | * |
|  | Mar. '20 SWR vs Nov. '20 SWB | 1 | 0.7072 | 0.17373 | 1.8923 | 0.013 | * |
|  | Mar. '20 SWR vs Nov. '20 SWR | 1 | 0.9515 | 0.2621 | 3.5519 | 0.004 | ** |
|  | Mar. '20 SWR vs Nov. '20 SMR | 1 | 0.3718 | 0.0885 | 0.8738 | 0.579 | n.s. |
|  | Mar. '20 SWR vs Nov. '21 SMB | 1 | 0.7472 | 0.14511 | 2.2067 | 0.005 | ** |
|  | Mar. '20 SWR vs Nov. '21 SWB | 1 | 0.674 | 0.15726 | 1.8661 | 0.01 | ** |
|  | Mar. '20 SWR vs Nov. '21 SWR | 1 | 0.7468 | 0.18602 | 2.2853 | 0.004 | ** |
|  | Mar. '20 SWR vs Nov. '21 SMR | 1 | 0.631 | 0.12703 | 1.6007 | 0.007 | ** |
|  | Mar. '20 SMR vs Nov. '20 SMB | 1 | 0.6952 | 0.15486 | 1.8323 | 0.002 | ** |
|  | Mar. '20 SMR vs Nov. '20 SWB | 1 | 0.7864 | 0.19314 | 2.1543 | 0.004 | ** |
|  | Mar. '20 SMR vs Nov. '20 SWR | 1 | 1.3905 | 0.34841 | 5.3471 | 0.003 | ** |
|  | Mar. '20 SMR vs Nov. '20 SMR | 1 | 0.476 | 0.1126 | 1.142 | 0.173 | n.s. |
|  | Mar. '20 SMR vs Nov. '21 SMB | 1 | 0.9141 | 0.17452 | 2.7485 | 0.001 | *** |
|  | Mar. '20 SMR vs Nov. '21 SWB | 1 | 0.8355 | 0.19124 | 2.3646 | 0.007 | ** |
|  | Mar. '20 SMR vs Nov. '21 SWR | 1 | 1.045 | 0.24677 | 3.2761 | 0.002 | ** |
|  | Mar. '20 SMR vs Nov. '21 SMR | 1 | 0.5682 | 0.11773 | 1.4678 | 0.015 | * |
|  | Nov. '20 SMB vs Nov. '20 SWB | 1 | 0.5486 | 0.16506 | 1.7792 | 0.011 | * |
|  | Nov. '20 SMB vs Nov. '20 SWR | 1 | 0.60488 | 0.22445 | 2.8941 | 0.003 | ** |
|  | Nov. '20 SMB vs Nov. '20 SMR | 1 | 0.4691 | 0.12642 | 1.3025 | 0.094 | n.s. |
|  | Nov. '20 SMB vs Nov. '21 SMB | 1 | 0.3978 | 0.09446 | 1.356 | 0.085 | n.s. |
|  | Nov. '20 SMB vs Nov. '21 SWB | 1 | 0.4285 | 0.12415 | 1.4175 | 0.058 | n.s. |
|  | Nov. '20 SMB vs Nov. '21 SWR | 1 | 0.6724 | 0.2006 | 2.5094 | 0.004 | ** |
|  | Nov. '20 SMB vs Nov. '21 SMR | 1 | 0.7336 | 0.16371 | 2.1533 | 0.001 | *** |
|  | Nov. '20 SWB vs Nov. '20 SWR | 1 | 0.79719 | 0.33516 | 4.5371 | 0.007 | ** |
|  | Nov. '20 SWB vs Nov. '20 SMR | 1 | 0.4542 | 0.14254 | 1.3299 | 0.068 | n.s. |
|  | Nov. '20 SWB vs Nov. '21 SMB | 1 | 0.5293 | 0.13807 | 1.9222 | 0.006 | ** |
|  | Nov. '20 SWB vs Nov. '21 SWB | 1 | 0.6777 | 0.21232 | 2.426 | 0.003 | ** |
|  | Nov. '20 SWB vs Nov. '21 SWR | 1 | 0.89608 | 0.29219 | 3.7153 | 0.001 | *** |
|  | Nov. '20 SWB vs Nov. '21 SMR | 1 | 0.7934 | 0.19677 | 2.4497 | 0.003 | ** |
|  | Nov. '20 SWR vs Nov. '20 SMR | 1 | 0.77402 | 0.27431 | 3.402 | 0.003 | ** |
|  | Nov. '20 SWR vs Nov. '21 SMB | 1 | 0.5746 | 0.17988 | 2.8513 | 0.003 | ** |
|  | Nov. '20 SWR vs Nov. '21 SWB | 1 | 0.5116 | 0.21852 | 2.7963 | 0.005 | ** |
|  | Nov. '20 SWR vs Nov. '21 SWR | 1 | 0.49566 | 0.25013 | 3.3357 | 0.017 | * |
|  | Nov. '20 SWR vs Nov. '21 SMR | 1 | 1.3797 | 0.35072 | 5.9417 | 0.002 | ** |
|  | Nov. '20 SMR vs Nov. '21 SMB | 1 | 0.4566 | 0.10801 | 1.4531 | 0.058 | n.s. |
|  | Nov. '20 SMR vs Nov. '21 SWB | 1 | 0.5996 | 0.16748 | 1.8106 | 0.012 | * |
|  | Nov. '20 SMR vs Nov. '21 SWR | 1 | 0.5785 | 0.17991 | 1.9744 | 0.024 | * |
|  | Nov. '20 SMR vs Nov. '21 SMR | 1 | 0.4259 | 0.10309 | 1.1494 | 0.201 | n.s. |
|  | Nov. '21 SMB vs Nov. '21 SWB | 1 | 0.5202 | 0.12773 | 1.9037 | 0.014 | * |
|  | Nov. '21 SMB vs Nov. '21 SWR | 1 | 0.7871 | 0.19696 | 3.1885 | 0.001 | *** |
|  | Nov. '21 SMB vs Nov. '21 SMR | 1 | 0.8052 | 0.15843 | 2.6357 | 0.001 | *** |
|  | Nov. '21 SWB vs Nov. '21 SWR | 1 | 0.29886 | 0.10997 | 1.2355 | 0.188 | n.s. |
|  | Nov. '21 SWB vs Nov. '21 SMR | 1 | 0.8766 | 0.20087 | 2.765 | 0.001 | *** |
|  | Nov. '21 SWR vs Nov. '21 SMR | 1 | 0.9203 | 0.22645 | 3.2202 | 0.001 | *** |
| ***Siderastrea radians*** | | | | | | | |
| **Multivariate Homogeneity of Groups Dispersions (betadisper)** | | | | | | | |
| **Variable** |  | **Df** | **Sum Sq** | **Mean Sq** | **F-value** | **p-value** | **sig.** |
| Location |  | 1 | 0.066811 | 0.066811 | 8.5834 | 0.006314 | ** |
| Timepoint |  | 2 | 0.040038 | 0.0200191 | 2.2554 | 0.1223 | n.s. |
| Location: Timepoint |  | 2 | 0.040038 | 0.0200191 | 2.2554 | 0.1223 | n.s. |
| **Permutational Multivariate Analysis of Variance (adonis)** | | | | | | | |
| **Variable** |  | **Df** | **SumOfSqs** | **R2** | **F-value** | **p-value** | **sig.** |
| Location |  | 1 | 1.2157 | 0.10299 | 3.9861 | 0.001 | *** |
| Timepoint |  | 2 | 1.4333 | 0.12142 | 2.3498 | 0.001 | *** |
| Location: Timepoint |  | 2 | 0.9205 | 0.07798 | 1.5092 | 0.018 | * |
| **Pairwise adonis** | | | | | | | |
| **Variable** | **Comparison** | **Df** | **SumOfSqs** | **R2** | **F-value** | **p-value** | **sig.** |
| Location | SM vs SW | 1 | 1.2157 | 0.10299 | 3.5592 | 0.001 | *** |
| Timepoint | Mar. '20 vs Nov. '20 | 1 | 0.9902 | 0.11736 | 2.9252 | 0.001 | *** |
|  | Mar. '20 vs Nov. '21 | 1 | 0.749 | 0.0959 | 2.0153 | 0.005 | ** |
|  | Nov. '20 vs Nov. '21 | 1 | 0.5114 | 0.0779 | 1.605 | 0.048 | * |
| Location: Timepoint | Mar. '20 SMB vs Mar. '20 SWB | 1 | 0.9919 | 0.23464 | 3.0657 | 0.002 | ** |
|  | Mar. '20 SMB vs Nov. '20 SWB | 1 | 1.211 | 0.24828 | 3.9635 | 0.001 | *** |
|  | Mar. '20 SMB vs Nov. '20 SMB | 1 | 0.9247 | 0.24891 | 2.6512 | 0.004 | ** |
|  | Mar. '20 SMB vs Nov. '21 SMB | 1 | 0.6027 | 0.14734 | 1.5553 | 0.02 | * |
|  | Mar. '20 SMB vs Nov. '21 SWB | 1 | 0.8062 | 0.23166 | 2.412 | 0.005 | ** |
|  | Mar. '20 SWB vs Nov. '20 SWB | 1 | 0.4873 | 0.13612 | 1.8909 | 0.018 | * |
|  | Mar. '20 SWB vs Nov. '20 SMB | 1 | 0.9521 | 0.30052 | 3.4371 | 0.005 | ** |
|  | Mar. '20 SWB vs Nov. '21 SMB | 1 | 0.8224 | 0.22012 | 2.5403 | 0.004 | ** |
|  | Mar. '20 SWB vs Nov. '21 SWB | 1 | 0.67082 | 0.24211 | 2.5556 | 0.015 | * |
|  | Nov. '20 SWB vs Nov. '20 SMB | 1 | 0.5729 | 0.17792 | 2.1643 | 0.009 | ** |
|  | Nov. '20 SWB vs Nov. '21 SMB | 1 | 0.6835 | 0.16969 | 2.2481 | 0.003 | ** |
|  | Nov. '20 SWB vs Nov. '21 SWB | 1 | 0.53138 | 0.17353 | 2.0996 | 0.027 | * |
|  | Nov. '20 SMB vs Nov. '21 SMB | 1 | 0.31583 | 0.11344 | 0.8957 | 0.542 | n.s. |
|  | Nov. '20 SMB vs Nov. '21 SWB | 1 | 0.44758 | 0.21292 | 1.6231 | 0.085 | n.s. |
|  | Nov. '21 SMB vs Nov. '21 SWB | 1 | 0.48241 | 0.1702 | 1.4357 | 0.053 | n.s. |
| **Branching *Porites* sp.** | | | | | | | |
| **Multivariate Homogeneity of Groups Dispersions (betadisper)** | | | | | | | |
| **Variable** |  | **Df** | **Sum Sq** | **Mean Sq** | **F-value** | **p-value** | **sig.** |
| Habitat |  | 1 | 0.000413 | 0.0004131 | 0.1536 | 0.701 | n.s. |
| Timepoint |  | 1 | 0.060088 | 0.060088 | 35.851 | 3.33E-05 | *** |
| Habitat: Timepoint |  | 3 | 0.37873 | 0.126244 | 36.605 | 2.56E-06 | *** |
| **Permutational Multivariate Analysis of Variance (adonis)** | | | | | | | |
| **Variable** |  | Df | SumOfSqs | R2 | **F-value** | **p-value** | **sig.** |
| Habitat |  | 1 | 0.4768 | 0.07415 | 1.1509 | 0.178 | n.s. |
| Timepoint |  | 1 | 0.5273 | 0.08201 | 1.2728 | 0.077 | n.s. |
| Habitat: Timepoint |  | 1 | 0.4542 | 0.07064 | 1.0963 | 0.258 | n.s. |

**Table S8.** Differential abundance of bacterial members using ALDEx2. Statistical comparisons of differentially abundant bacteria were made between coral species, and within-species comparisons of timepoint, habitat, site, habitat:timepoint, and site:timepoint. Only significant differential abundance enrichments by GLM anova (ALDEx2) are shown (p < 0.05). Adjusted p-values are Benjamini-Hochberg adjusted for post-hoc comparisons. Mar. = March, Nov. = November, SM = Santa Martha, SW = Spaanse Water, B = Bay, R = Reef.

| **Coral species** | | | | | |
| --- | --- | --- | --- | --- | --- |
| **Feature** | **Factor** | **Enriched Group** | **F (ALDEx2)** | **p-value (unadj.)** | **p-value (adj.)** |
| Class Bacteroidia | Species | *S. radians* | 26.61965 | 1.79E-09 | <0.001 |
| ***Siderastrea siderea*** | | | | | |
| **Feature** | **Factor** | **Enriched Group** | **F (ALDEx2)** | **p-value (unadj.)** | **p-value (adj.)** |
| Sva0996 marine group | Timepoint | Mar. '20 | 11.222604 | 2.67E-04 | 0.013 |
| Roseimarinus |  | Mar. '20 | 11.856732 | 5.28E-04 | 0.016 |
| Family Saprospiraceae |  | Mar. '20 | 7.476574 | 3.12E-03 | 0.049 |
| Ekhidna |  | Mar. '20 | 10.849303 | 3.28E-04 | 0.014 |
| Muricauda |  | Mar. '20 | 7.772552 | 1.18E-03 | 0.036 |
| Winogradskyella |  | Mar. '20 | 8.369482 | 2.86E-03 | 0.044 |
| Family Caldilineaceae |  | Mar. '20 | 13.502016 | 3.66E-05 | 0.005 |
| Fusibacter |  | Mar. '20 | 11.624151 | 4.85E-04 | 0.015 |
| Kingdom Bacteria |  | Mar. '20 | 8.296199 | 1.89E-03 | 0.035 |
| Class Gracilibacteria |  | Mar. '20 | 8.753828 | 1.28E-03 | 0.032 |
| Class OM190 |  | Mar. '20 | 11.99282 | 8.06E-05 | 0.008 |
| Phycisphaera |  | Mar. '20 | 8.436249 | 1.08E-03 | 0.031 |
| Class Pla3 lineage |  | Mar. '20 | 9.182327 | 1.82E-03 | 0.034 |
| Blastopirellula |  | Mar. '20 | 11.026544 | 1.98E-04 | 0.012 |
| Family Pirellulaceae |  | Mar. '20 | 8.442071 | 2.04E-03 | 0.040 |
| Pir4 lineage |  | Mar. '20 | 8.64758 | 1.26E-03 | 0.032 |
| Order Planctomycetales |  | Mar. '20 | 9.889295 | 1.14E-03 | 0.026 |
| Family Rubinisphaeraceae |  | Mar. '20 | 10.011219 | 1.15E-03 | 0.026 |
| Class Alphaproteobacteria |  | Mar. '20 | 8.116613 | 1.55E-03 | 0.036 |
| Order BD7-8 |  | Mar. '20 | 8.477168 | 3.19E-03 | 0.041 |
| Class Gammaproteobacteria |  | Mar. '20 | 7.581515 | 2.41E-03 | 0.047 |
| Alteromonas |  | Nov. '21 | 8.985392 | 4.16E-04 | 0.021 |
| Pseudoalteromonas |  | Nov. '21 | 9.07222 | 2.56E-04 | 0.018 |
| Psychrobacter |  | Nov. '21 | 13.958058 | 2.19E-04 | 0.009 |
| Family Chloroflexaceae | Habitat | reef | 0.9622874 | 2.21E-06 | 0.002 |
| Endozoicomonas |  | reef | 0.5160516 | 1.98E-04 | 0.047 |
| Candidatus Amoebophilus |  | bay | -0.6488671 | 5.59E-05 | 0.022 |
| Synechococcus CC9902 |  | bay | -0.600171 | 7.01E-06 | 0.005 |
| Candidatus Amoebophilus | Site | SMB | 10.673637 | 2.64E-05 | 0.004 |
| Robertkochia |  | SMB | 12.047263 | 4.01E-05 | 0.004 |
| Synechococcus CC9902 |  | SMB | 6.934764 | 7.16E-04 | 0.031 |
| Family Kiloniellaceae |  | SMB | 7.105199 | 1.43E-03 | 0.040 |
| Family Chloroflexaceae |  | SMR | 14.216101 | 3.30E-06 | 0.001 |
| Family Desulfocapsaceae |  | SWB | 11.175197 | 9.34E-05 | 0.007 |
| Thiohalophilus |  | SWB | 7.812345 | 8.17E-04 | 0.028 |
| Family Thiotrichaceae |  | SWB | 8.397262 | 3.69E-04 | 0.018 |
| Alteromonas |  | SWR | 6.68249 | 5.86E-04 | 0.031 |
| Algicola |  | SWR | 7.696836 | 3.28E-04 | 0.020 |
| Roseimarinus | Habitat: Timepoint | Mar. '20 bay | 5.700389 | 3.22E-03 | 0.038 |
| Candidatus Amoebophilus |  | Mar. '20 bay | 5.009613 | 2.24E-03 | 0.036 |
| Family Caldilineaceae |  | Mar. '20 bay | 5.919037 | 3.29E-04 | 0.017 |
| Fusibacter |  | Mar. '20 bay | 5.237959 | 1.36E-03 | 0.032 |
| Class OM190 |  | Mar. '20 bay | 5.241348 | 3.08E-03 | 0.034 |
| Blastopirellula |  | Mar. '20 bay | 4.900692 | 1.11E-03 | 0.035 |
| Order Planctomycetales |  | Mar. '20 bay | 5.150399 | 1.86E-03 | 0.037 |
| Family Rubinisphaeraceae |  | Mar. '20 bay | 5.339524 | 1.13E-03 | 0.030 |
| Phylum Proteobacteria |  | Mar. '20 reef | 5.140315 | 1.29E-03 | 0.034 |
| Alteromonas |  | Nov. '20 reef | 4.739961 | 9.99E-04 | 0.035 |
| Pseudoalteromonas |  | Nov. '21 bay | 4.190613 | 1.43E-03 | 0.049 |
| Psychrobium |  | Nov. '21 bay | 6.099069 | 1.34E-03 | 0.025 |
| Cutibacterium |  | Nov. '21 reef | 5.071382 | 3.86E-03 | 0.045 |
| Porphyromonas |  | Nov. '21 reef | 5.246063 | 5.83E-04 | 0.025 |
| Family Chloroflexaceae |  | Nov. '21 reef | 9.181385 | 2.53E-05 | 0.002 |
| Endozoicomonas |  | Nov. '21 reef | 6.036354 | 1.00E-04 | 0.012 |
| Psychrobacter |  | Nov. '21 reef | 8.621241 | 2.30E-05 | 0.003 |
| Class Subgroup 22 | Site: Timepoint | Mar. '20 SMB | 3.67448 | 2.75E-03 | 0.025 |
| Roseimarinus |  | Mar. '20 SMB | 5.694413 | 7.97E-06 | 0.001 |
| Candidatus Amoebophilus |  | Mar. '20 SMB | 4.032743 | 1.62E-04 | 0.006 |
| Family Cyclobacteriaceae |  | Mar. '20 SMB | 2.741785 | 4.92E-03 | 0.050 |
| Robertkochia |  | Mar. '20 SMB | 4.057399 | 1.10E-03 | 0.014 |
| Family Bacteriovoracaceae |  | Mar. '20 SMB | 3.007954 | 4.96E-03 | 0.043 |
| Family Anaerolineaceae |  | Mar. '20 SMB | 3.575108 | 1.37E-03 | 0.019 |
| Family Caldilineaceae |  | Mar. '20 SMB | 3.31479 | 2.01E-03 | 0.025 |
| Class Thermodesulfovibrionia |  | Mar. '20 SMB | 3.022939 | 5.50E-03 | 0.044 |
| Blastopirellula |  | Mar. '20 SMB | 3.906201 | 9.03E-04 | 0.014 |
| Rhodopirellula |  | Mar. '20 SMB | 3.602128 | 1.52E-03 | 0.020 |
| Order Planctomycetales |  | Mar. '20 SMB | 3.131172 | 3.62E-03 | 0.035 |
| Family Rubinisphaeraceae |  | Mar. '20 SMB | 3.360586 | 2.96E-03 | 0.028 |
| Alcaligenes |  | Mar. '20 SMB | 2.862797 | 5.25E-03 | 0.048 |
| Gammaproteobacteria Incertae Sedis |  | Mar. '20 SMB | 3.654463 | 9.58E-04 | 0.016 |
| Halomonas |  | Mar. '20 SMB | 3.11705 | 2.87E-03 | 0.032 |
| Microbulbifer |  | Mar. '20 SMB | 2.961848 | 5.13E-03 | 0.046 |
| Family Nitrincolaceae |  | Mar. '20 SMB | 2.889217 | 4.98E-03 | 0.048 |
| Oleiphilus |  | Mar. '20 SMB | 3.417973 | 3.81E-03 | 0.032 |
| Phylum Proteobacteria |  | Mar. '20 SMB | 3.415729 | 3.24E-03 | 0.026 |
| Oceanotoga |  | Mar. '20 SMB | 3.04379 | 4.65E-03 | 0.037 |
| Lewinella |  | Mar. '20 SMR | 3.277902 | 1.88E-03 | 0.026 |
| Family Chloroflexaceae |  | Mar. '20 SMR | 4.806276 | 1.23E-04 | 0.003 |
| Class OM190 |  | Mar. '20 SMR | 2.997992 | 3.97E-03 | 0.041 |
| Order BD7-8 |  | Mar. '20 SMR | 3.943475 | 4.45E-04 | 0.009 |
| Order WCHB1-41 |  | Mar. '20 SMR | 3.744929 | 1.21E-03 | 0.018 |
| Ekhidna |  | Mar. '20 SWB | 3.108428 | 2.03E-03 | 0.029 |
| Fusibacter |  | Mar. '20 SWB | 3.27101 | 2.06E-03 | 0.025 |
| Kingdom Bacteria |  | Mar. '20 SWR | 2.964845 | 2.61E-03 | 0.034 |
| Endozoicomonas |  | Mar. '20 SWR | 3.29007 | 7.06E-04 | 0.017 |
| Family Desulfocapsaceae |  | Nov. '20 SWB | 4.189736 | 4.74E-04 | 0.009 |
| Thiogranum |  | Nov. '20 SWB | 3.645094 | 2.80E-03 | 0.026 |
| Family Thiotrichaceae |  | Nov. '20 SWB | 3.463346 | 2.18E-03 | 0.024 |
| Profundimonas |  | Nov. '20 SWR | 2.970955 | 6.02E-03 | 0.046 |
| Psychrobium |  | Nov. '21 SMB | 3.965761 | 1.60E-03 | 0.016 |
| Porphyromonas |  | Nov. '21 SMR | 3.567742 | 6.64E-04 | 0.014 |
| Prosthecochloris |  | Nov. '21 SMR | 2.995325 | 4.24E-03 | 0.041 |
| Streptococcus |  | Nov. '21 SMR | 2.999515 | 1.38E-03 | 0.026 |
| Erythrobacter |  | Nov. '21 SMR | 3.306192 | 2.19E-03 | 0.026 |
| Thalassotalea |  | Nov. '21 SWB | 2.753102 | 3.59E-03 | 0.043 |
| Algicola |  | Nov. '21 SWB | 4.352093 | 5.33E-05 | 0.003 |
| Marinomonas |  | Nov. '21 SWB | 4.43805 | 1.78E-04 | 0.005 |
| Thalassolituus |  | Nov. '21 SWB | 3.418642 | 1.49E-03 | 0.021 |
| Alteromonas |  | Nov. '21 SWR | 10.358018 | 2.03E-09 | 1.40E-06 |
| Pseudoalteromonas |  | Nov. '21 SWR | 4.672483 | 2.99E-05 | 0.002 |
| Psychrosphaera |  | Nov. '21 SWR | 4.845108 | 1.69E-05 | 0.001 |
| Psychrobacter |  | Nov. '21 SWR | 5.348188 | 5.80E-05 | 0.002 |
| Oleibacter |  | Nov. '21 SWR | 3.103066 | 9.30E-03 | 0.047 |
| ***Siderastrea radians*** | | | | | |
| **Feature** | **Factor** | **Enriched Group** | **F (ALDEx2)** | **p-value (unadj.)** | **p-value (adj.)** |
| Alteromonas | Timepoint | Nov. '21 | 21.2619 | 1.93E-06 | 0.002 |
| Marinomonas |  | Nov. '21 | 15.11322 | 7.70E-05 | 0.020 |
| Family Flavobacteriaceae | Location | SWB | 15.35353 | 3.78E-04 | 0.045 |
| Thiogranum |  | SWB | 20.43716 | 5.87E-04 | 0.035 |
| Family Thiotrichaceae |  | SWB | 22.81862 | 1.72E-04 | 0.019 |
| Kingdom Bacteria | Location: Timepoint | Mar. '20 SMB | 5.320058 | 9.31E-04 | 0.035 |
| Class Bacteroidia |  | Mar. '20 SWB | 4.556046 | 1.22E-03 | 0.048 |
| Family Desulfocapsaceae |  | Mar. '20 SWB | 6.547666 | 1.05E-03 | 0.026 |
| Thiogranum |  | Mar. '20 SWB | 7.396649 | 2.64E-04 | 0.014 |
| Thiohalophilus |  | Mar. '20 SWB | 6.892547 | 7.07E-04 | 0.022 |
| Family Thiotrichaceae |  | Mar. '20 SWB | 5.34274 | 1.87E-03 | 0.040 |
| Family Flavobacteriaceae |  | Nov. '20 SWB | 4.565206 | 1.31E-03 | 0.048 |
| Tistlia |  | Nov. '21 SMB | 5.139026 | 1.17E-03 | 0.039 |
| Order Thalassobaculales |  | Nov. '21 SWB | 5.650121 | 4.36E-04 | 0.024 |
| Alteromonas |  | Nov. '21 SWB | 9.821751 | 1.34E-05 | 0.003 |
| Psychrosphaera |  | Nov. '21 SWB | 5.623724 | 1.77E-03 | 0.041 |
| Vibrio |  | Nov. '21 SWB | 6.801088 | 1.62E-04 | 0.012 |
| Marinomonas |  | Nov. '21 SWB | 8.564185 | 3.97E-04 | 0.011 |
| Thalassolituus |  | Nov. '21 SWB | 6.565392 | 9.81E-04 | 0.026 |
| **Branching *Porites* sp.** | | | | | |
| No significant differentially abundant bacteria | | | | | |
